# Supplementary material for: Multidrug Resistance (MDR) and Collateral Sensitivity in Bacteria, with Special Attention to Genetic and Evolutionary Aspects and to the Perspectives of Antimicrobial Peptides—A Review
Source: Pathogens. 2020 Jun 29;9(7):522. doi: 10.3390/pathogens9070522 (PMC7399985; doi:10.3390/pathogens9070522)
Supplement: Supplementary file 1 [file pathogens-09-00522-s001.pdf]

SUPPLEMENTARY MATERIAL 1 /FODOR ET AL

Zoonic and veterinary pathogen candidates for the "ESCAPE Club"

S1.1 *Escherichia coli*

Commensal strains of E. coli, as versatile residents of the lower intestine, are also repeatedly challenged by antimicrobial pressures during the lifetime of their host. Consequently, commensal strains acquire resistance genes, and/or develop resistant mutants in order to survive and maintain microbial homeostasis in the lower intestinal tract. Commensal *E. coli* strains are regarded as indicators of the antimicrobial load of their hosts. The recent review (Szmolka, A. and Nagy, B. 2013) described the historical background of the origin, appearance and transfer mechanisms of antimicrobial resistance genes into an original animal - commensal intestinal *E. coli* with comparative information on their pathogenic counterparts. The most efficient mechanism used by E. coli against different antimicrobial-based efflux pumps and mobile resistance mechanisms carried by plasmids and/or mobile genetic elements is known. For a while, these mechanisms cannot protect *E. coli* against fabclavine (Fodor L. et al., in preparation). The emergence of hybrid plasmids, both resistance and virulent, among *E. coli* is of additional public concern. Co-existence and co-transfer of these "bad genes" in this huge and most versatile in vivo compartment may represent increased public health risk in the future. The significance of MDR commensal E. coli seems to be highest in the food animal industry, which may function as a reservoir for intra- and interspecific exchange, and a source for the spread of MDR determinants through contaminated food to humans. Thus, the potential of MDR occurring in these commensal bacteria living in animals used as sources of food (as meat, eggs, milk) should be a concern from the aspect of public health, and it needs to be continuously monitored in the future by using the toolkit of molecular genetics [8]. In fact, that pessimistic theory has been demonstrated. The first pilot study on the prevalence of verocytotoxin-producing (VTEC) E. coli and of MDR/ESBL E. coli in illegally imported food products of animal origin, suggests that these strains could represent reservoirs for dissemination of potentially new types of pathogenic and MDR *E. coli* in Europe [287] (Card et al., 2017). The latest British simulation experiments have supported this prognosis [288] (Dame and Shapiro, 1979). The authors developed an in vitro chemostat system to approximate the chicken caecal microbiota, simulated colonization by an MDR *Salmonella* pathogen and examined the dynamics of transfer of its MDR plasmid, harboring several genes, including the extended-spectrum beta-lactamase blaCTX-M1. They also evaluated the impact of cefotaxime administration on plasmid transfer and microbial diversity. Bacterial community profiles, obtained by culture-independent methods, showed that *Salmonella* inoculation resulted in no significant changes to bacterial community alpha and beta diversity, whereas administration of cefotaxime caused significant alterations to both measures of diversity, which largely recovered. MDR plasmid transfer from Salmonella to commensal E. coli was demonstrated by the polymerase chain reaction (PCR) technique, and whole-genome sequencing of isolates purified from agar plates containing cefotaxime. The transfer occurred in seven *E. coli* sequence types at high rates, even in the absence of cefotaxime, with resistant strains isolated within 3 days [288] (Dame and Shapiro, 1979).

REFERENCES TO S1.1 *Escherichia coli*

.

Card, R. M., Cawthraw, S. A., Nunez-Garcia, J., Ellis, R. J., Kay, G., Pallen, M. J., Woodward, M. J. and Anjum, M. F. (2017). An *in vitro* chicken gut model demonstrates transfer of a multidrug resistance plasmid from Salmonella to commensal *Escherichia coli*. *MBio*. 8, e00777-17. DOI: 10.1128/mBio.00777-17.

Dame, J. B. and Shapiro, B. M. (1979). Lipid and lipopolysaccharide composition of *Escherichia coli* surface-altered mutants selected for resistance to levallorphan, tetracaine, and polymixin. *J. Bacteriol*. 137, 1043-1047.

Szmolka, A. and Nagy, B. (2013). Multidrug resistant commensal *Escherichia coli* in animals and its impact for public health. *Front Microbiol.* 4, 258.

Ibrahim, M. E., Bilal, N. E.and Hamid, M. E. (2012). Increased multi-drug resistant *Escherichia coli* from hospitals in Khartoum state, Sudan. *Afr. Health Sci*. 12, 368-375. DOI: 10.4314/ahs.v12i3.19

S1.2 *Francisella tularensis*

Francisella tularensis is a fastidious, Gram-negative bacterium, a highly contagious zoonotic agent, and the causative agent of the fatal disease, tularemia. **Tularemia** may occur in six well-recognized clinical forms in humans: ulceroglandular; glandular; oculoglandular; oropharyngeal; pneumonic; and typhoid, or septicemic, tularemia. The *F. tularensis* subsp. holarctica (type B) (Gyuranecz et al., 2010) can be found throughout the Northern Hemisphere and is the only endemic subspecies found in Europe, (Gyuranecz et al., 2012). Lagomorphs, rodents, European brown hares (*Lepus europaeus*), and voles (*Microtus arvalis*), serve as the primary mammalian reservoir hosts. The annual number of tularemia cases in humans is well correlated with the yearly biologic cycle (March-February) for hares and hematophagous arthropods, such as ticks, which play a role as vectors and hosts [285] (Kreizinger et al., 2013). The antibiotics of choice in the treatment of tularemia are aminoglycosides, quinolones, chloramphenicol, or tetracyclines.

Fortunately, enough, there has been no sign of the occurrence of multi-drug resistant *F. tularensis* strains, but resistance to the same antibiotics used in its treatment are known in other bacteria, so acquired resistance could be forecast. Furthermore, aminoglycosides, quinolones, chloramphenicol, and tetracyclines are important, bearing in mind the side effects and probability to replace them. Therefore, it is important to be informed about the general picture of the antibiotic susceptibility of local populations. A recent screen in Hungary provided a satisfying result (Nagy et al., 2015). Twenty-nine *F. tularensis* strains isolated between 2003 and 2010 from free-ranging European brown hares, and a captive patas monkey (*Erythrocebus patas*), were collected from different parts of Hungary.

Each isolate belonged to *F. tularensis* subsp. *holarctica*, phylogenetic group B.13. Each strain was susceptible to those antibiotics which have commonly been used in therapy, such as aminoglycosides, gentamicin, streptomycin, tetracycline, doxycycline, quinolones, ciprofloxacin, levofloxacin, and chloramphenicol, and in addition tigecycline and rifampicin. Naturally, they were resistant to erythromycin and linezolid.

REFERENCES TO S1.2 *Francisella tularensis*

283. Gyuranecz, M., Erdélyi, K., Fodor, L., Jánosi, K., Szépe, B., Füleki, M., Szoke, I., Dénes, B. and Makrai, L. (2010). Characterization of *Francisella tularensis* strains, comparing their carbon source utilization. *Zoonoses Pub. Health*. 57, 417-422. DOI:10.1111/j.1863-2378.2009.01238. x.
284. Gyuranecz, M., Reiczigel, J., Krisztalovics, M. L., Monse, L., Szabóné, G. K., Szilágyi, A., Szépe, B., Makrai, L., Magyar, T., Bhide, M. and Erdélyi, K. (2012). Factors influencing emergence of tularemia, Hungary, 1984–2010. *Emerg. Infect. Dis*.18, 1379. DOI: 10.3201/eid1808.111826.
285. Kreizinger, Z., Makrai, L., Helyes, G., Magyar, T., Erdélyi, K. and Gyuranecz, M. (2012). Antimicrobial susceptibility of *Francisella tularensis* subsp. *holarctica* strains from Hungary, Central Europe. *J. Antimicrob. Chemother*. 68, 370-373. DOI: 10.1093/jac/dks399.
286. Nagy, B., Szmolka, A., Smole Možina, S., Kovač, J., Strauss, A., Schlager, S., Beutlich, J., Appel, B., Lušicky, M, Aprikian, P., Pászti, J., Tóth, I., Kugler, R. and Wagner, M. (2015). Virulence and antimicrobial resistance determinants of verotoxigenic *Escherichia coli* (VTEC) and of multidrug-resistant *E. coli* from foods of animal origin illegally imported to the EU by flight passengers. *Int. J. Food Microbiol*. 209, 52-59. DOI: 10.1016/j.ijfoodmicro.2015.06.026.

S1.3. *Mycoplasma bovis*

Mycoplasma bovis is a worldwide pathogen that is the causative agent of pneumonia, mastitis, arthritis, and a variety of other symptoms in cattle [262] (Citti and Blanchard, 2013). As a result, it is responsible for significant economic losses [263] (Taylor-Robinson and Bebear, 1997). The pathogens in the *Mycoplasm*a species are members of the class Mollicutes and comprise the simplest life form that can replicate independently from the host. *Mycoplasm*a spp. have no cell wall, and they have a limited number of metabolic pathways. The greatly reduced genome size and coding capacity of Mycoplasma spp. makes them a good model for genetic studies. *Mycoplasma* spp. are rather fast-evolving bacteria, pathogenic against humans and animals. However, their importance is often underestimated. *Mycoplasma bovis* is a major cause of calf pneumonia, mastitis, and arthritis, and is intrinsically resistant to antibiotics acting on cell wall or folate synthesis [262] (Citti and Blanchard, 2013), but the antimicrobial protein synthesis inhibitor classes are active against it [264] (Lysnyansky and Ayling, 2016). Tetracyclines and spectinomycin primarily bind to the 30S subunit of the ribosome, whereas macrolides, lincosamides, phenicols, and pleuromutilins are mycoplasmastatic antibiotics acting on the 50S ribosomal subunit, preventing the mechanisms of transpeptidation and translation [265] (Piddock, 1999).

Expanded-spectrum fluoroquinolones, such as enrofloxacin, danofloxacin, and marbofloxacin, have anti-mycoplasmacidal effects by acting on topoisomerases that inhibit the DNA synthesis of bacteria [266] (Gautier-Bouchardon et al., 2014).

Among the few antimicrobials licensed for the treatment of *M. bovis*, there is increasing evidence for resistance [254, 265-268] (Piddock, 1999; Courvalin, 2006; Sato et al., 2013; Gautier-Bouchardon et al., 2014; Heuvelink et al., 2016).

As for the genetic background, a point mutation in the *par*C gene resulted in decreased susceptibility to fluoroquinolones in M. bovis [265] (Piddock, 1999), and amino acid substitutions in GyrA and ParC resulted in fluoroquinolone-resistant phenotypes [269] (Amram et al., 2015). Surprisingly enough, 16S rRNA gene mutations have been associated with decreased susceptibility to tetracycline in M. bovis [270] (Kong et al., 2016). *M. bovis* was detected in 32/45 bovine respiratory infection outbreaks at beef farms in 8 provinces in China [271] (Sulyok et al., 2014). The isolates were susceptible, or had medium sensitivity, to ciprofloxacin, enrofloxacin, and doxycycline, but 13 of the 32 were resistant to macrolides. A point mutation at the 23 *rrn*A operon in domain V of 23S rRNA seems to be responsible for the macrolide resistance phenotype in M. bovis [271] (Sulyok et al., 2014). Antibiotic susceptibility profiles of *M. bovis* strains isolated from cattle in Hungary were determined [272] (Sulyok et al., 2017). The growth of many *M. bovis* strains was not inhibited by gentamicin, spectinomycin, florfenicol or lincomycin. The most effective antibiotics tested *in vitro* were the fluoroquinolones: danofloxacin, enrofloxacin, and marbofloxacin. But there were 3 of the 35 Hungarian field strains for which the fluoroquinolone MICs were high [272] (Sulyok et al., 2017). Fluoroquinolone-resistant mutants were also selected *in vitro* for danofloxacin, enrofloxacin, and marbofloxacin and each showed complete cross-resistance with the others. The respective mutations responsible for high macrolide, lincomycin, florfenicol, and pleuromutilin antibiotic MICs were mapped into genes encoding 23S rRNA [273] (Mock and Fouet, 2001).

Despite evidence that this is a major cause of calf pneumonia, mastitis and arthritis, keratoconjunctivitis, otitis media and genital disorders that may result in infertility and abortion, .especially in cattle, no commercial vaccines exist for *Mycoplasma bovis*, (Nicholas et al., 2009). Antimicrobial resistance is increasing; diagnostic and antimicrobial sensitivity testing needs to be improved; and a pen-side test would facilitate more rapid diagnosis and implementation of treatment with antimicrobials. More data on host susceptibility, stress factors, immune response and infectious dose levels are required, (Calcutt et al., 2018).

REFERENCES TO S1.3. *Mycoplasma bovis*

261. Nicholas, R. A. J. and Ayling, R. D. (2003). Mycoplasma bovis: disease, diagnosis, and control. *Res. Vet. Sci.* 74, 105-112. DOI: 10.1016/S0034-5288(02)00155-8.
262. Citti, C. and Blanchard, A. (2013). Mycoplasmas and their host: emerging and re-emerging minimal pathogens. *Trends Microbiol*. 21, 196-203. DOI: 10.1016/j.tim.2013.01.003.
263. Taylor-Robinson, D. and Bebear, C. (1997). Antibiotic susceptibilities of mycoplasmas and treatment of mycoplasmal infections. *J. Antimicrob. Chemother*. 40, 622-630. DOI: 10.1093/jac/40.5.622.
264. Lysnyansky, I. and Ayling, R. D. (2016). *Mycoplasma bovis*: mechanisms of resistance and trends in antimicrobial susceptibility. *Front. Microbiol*. 7, 595. DOI: 10.3389/fmicb.2016.00595.
265. Piddock, L. J. (1999). Mechanisms of fluoroquinolone resistance: an update 1994-1998. *Drugs* 58, 11-18.

266. Gautier-Bouchardon, A. V., Ferré, S., Le Grand, D., Paoli, A., Gay, E. and Poumarat, F. (2014). Overall decrease in the susceptibility of *Mycoplasma bovis* to antimicrobials over the past 30 years in France. *PLoS One.* 9, 87672. DOI: 10.1371/journal.pone.0087672.
267. Heuvelink, A., Reugebrink, C. and Mar, J. (2016). Antimicrobial susceptibility of *Mycoplasma bovis* isolates from veal calves and dairy cattle in the Netherlands. *Vet. Microbiol*. 189, 1-7. DOI: 10.1016/j.vetmic.2016.04.012.
268. Sato, T., Okubo, T., Usui, M., Higuchi, H. and Tamura, Y. (2013). Amino acid substitutions in GyrA and ParC are associated with fluoroquinolone resistance in *Mycoplasma bovis* isolates from Japanese dairy calves. *J. Vet. Med. Sci*. 75, 1063-1065. DOI: 10.1292/jvms.12-0508.
269. Amram, E., Mikula, I., Schnee, C., Ayling, R. D., Nicholas, R. A., Rosales, R. S., Harrus, S. and Lysnyansky, I. (2015). 16S rRNA gene mutations associated with decreased susceptibility to tetracycline in *Mycoplasma bovis*. *Antimicrob. Agents Chemother*. 59, 796-802. DOI: 10.1128/AAC.03876-14.
270. Kong, L. C., Gao, D., Jia, B. Y., Wang, Z., Gao, Y. H., Pei, Z. H., Liu, S. M., Xin, J. Q. and Ma, H. X. (2016). Antimicrobial susceptibility and molecular characterization of macrolide resistance of *Mycoplasma bovis* isolates from multiple provinces in China. *J. Vet. Med. Sci*. 78, 293-296. DOI: 10.1292/jvms.15-0304.
271. Sulyok, K. M., Kreizinger, Z., Fekete, L., Hrivnák, V., Magyar, T., Jánosi, S., Schweitzer, N., Turcsányi, I., Makrai, L., Erdélyi, K. and Gyuranecz, M. (2014). Antibiotic susceptibility profiles of *Mycoplasma bovis* strains isolated from cattle in Hungary, Central Europe. *BMC Vet. Res*. 10, 256. DOI: 10.1186/s12917-014-0256-x.
272. Sulyok, K. M., Kreizinger, Z., Wehmann, E., Lysnyansky, I., Bányai, K., Marton. S., Jerzsele, Á., Rónai, Z., Turcsányi, I., Makrai, L., Jánosi, S., Nagy, S. Á. and Gyuranecz, M. (2017). Mutations associated with decreased susceptibility to seven antimicrobial families in field and laboratory-derived *Mycoplasma bovis* strains. *Antimicrob. Agents Chemother*. 61, e01983-16. DOI: 10.1128/AAC.01983-16.
273. Mock, M. and Fouet, Á. (2001). Anthrax. *Annu. Rev. Microbiol*. 55, 647-671. DOI: 10.1146/annurev.micro.55.1.647
[274] Nicholas, R. A, Ayling, R. D. and McAuliffe, L. (2009). Vaccines for Mycoplasma diseases in animals and man. *J. Comp. Pathol.* 140, 85-96. doi: 10.1016/j.jcpa.2008.08.004. Epub 2008 Dec 25. Review. PMID: 19111314
[275] Calcutt, M.J., Lysnyansky, I., Sachse, K., Fox, L. K., Nicholas, R. A. J. and Ayling, R. D. (2018). Gap analysis of Mycoplasma bovis disease, diagnosis and control: An aid to identify future development requirements. *Transbound Emerg. Dis.* 1, 91-109. doi: 10.1111/tbed.12860. Epub 2018 Mar 27.

.

S1.4. *Bacillus anthracis*

Bacillus anthracis, the bacterium of Koch and Pasteur, is the etiologic agent of anthrax, a common disease of livestock and, occasionally, of humans. It is the only obligate pathogen within the genus Bacillus. Bacillus anthracis is a Gram-positive, endospore-forming, rod-shaped, bacterium, (Wikipedia). It causes extremely severe zoonoses, posing a serious threat to both public and animal health [274] (Inglesby et al., 2002). Bacillus anthracis belongs to the B. cereus group of bacteria. Infection with this bacterium can occur through the skin, gastrointestinal tract,

or respiratory apparatus, following contact, ingestion, or inhalation of spores, respectively. The fluoroquinolones (FQs) are first-line antibiotics for the treatment of B. anthracis infection, and as a result, FQ resistance is a major concern for medical treatment following anthrax as a bioterrorism tool [275] (Ruiz, 2003). FQs act as broad-spectrum bactericidal antibiotics by inhibiting type II DNA topoisomerases, DNA gyrases (GyrA and GyrB), and type IV DNA topoisomerases (ParC and ParE). The mechanism responsible for FQ resistance has been well documented with bacteria, in which frequent mutations of topoisomerase genes have been identified in the designated quinolone resistance-determining region (QRDR) [276] (Aldred et al., 2012). A recent detailed study to determine the basis for quinolone action and resistance was undertaken by Alfred et al. [277] (Markham and Neyfakh, 2001). They compared the B. anthracis topoisomerase IV of the wild-type, and the GrlA (S81F) and GrlA (S81Y) of the quinolone-resistant mutants, in the presence or absence of quinolones and a related quinazolinedione, to determine the effects on these enzymes. Ser81 is believed to anchor a water-Mg (2+) bridge that coordinates quinolones to the enzyme through the C3/C4 keto acid. Consistent with this hypothesized bridge, ciprofloxacin required increased Mg (2+) concentrations to support DNA cleavage by GrlA (S81F) topoisomerase IV.

The three enzymes displayed similar catalytic activities in the absence of drugs. However, the resistant mutations decreased the affinity of topoisomerase IV for ciprofloxacin and other quinolones, diminished quinolone-induced inhibition of DNA religation and reduced the stability of the enzyme-quinolone-DNA ternary complex. Wild-type DNA cleavage levels were generated by mutant enzymes at high quinolone concentrations, suggesting that increased drug potency could overcome resistance. 8-Methyl-quinazoline-2,4-dione, which lacks the quinolone keto acid, and presumably does not require the water-Mg (2+) bridge to mediate protein interactions, was more potent than other quinolones against wild-type topoisomerase IV. Moreover, it maintained high potency and efficacy against the mutant enzymes, effectively inhibited DNA religation and formed stable ternary complexes.

In fact, reports have also suggested a possible contribution of multi-drug efflux pumps to FQ resistance in B. anthracis [278] (Serizawa et al., 2010). The genome-wide screening for novel genetic variations associated with ciprofloxacin resistance in B. anthracis resulted in the discovery of 2 strains showing resistance, or intermediate resistance, to ciprofloxacin (CIP) by a stepwise selection procedure with increasing CIP concentrations [279, 280] (Raskó et al., 2011; Kreizinger et al., 2016). Fifteen genetic variations were identified between the parental and CIP-resistant strains by next-generation sequencing. Nonsynonymous mutations in the quinolone resistance-determining region (QRDR)

of the type II DNA topoisomerase were identified in the resistant strain, but not in the intermediate-resistant strain. The authors discovered a novel “mutation hot spot” (GBAA0834) that leads to the increased expression of multi-drug efflux systems for CIP resistance. Such disruptive mutations appear to be more easily acquired than those in an essential gene, such as that encoding type II DNA topoisomerase. Such an intermediate-resistant phenotype could increase a cell population under CIP-selective pressure and might promote the emergence of highly resistant isolates [279] (Raskó et al., 2011).

The susceptibility of 29 *B. anthracis* bovine strains, collected in Hungary between 1933 and 2014 was tested against 10 antibiotics with commercially available minimum inhibitory concentration (MIC) test strips [281] (Pilo et al., 2011). All strains were susceptible to

amoxicillin, ciprofloxacin, clindamycin, doxycycline, gentamicin, penicillin, rifampicin, and vancomycin. Intermediate susceptibility to erythromycin and cefotaxime was detected in 17.2% (5/29) and 58.6% (17/29) of the strains, respectively. Correlations were not observed between the isolation date, location, host species, genotype, and antibiotic susceptibility profile of the strain. A similar study in Cameroon showed that Bovine B. anthracis isolates from there showed a strong homogeneity, and they belong, together with strains from Chad, to a cluster Aβ, which appears to be predominant in western Africa [282] (WHO, 2007). However, one strain that belongs to a newly defined clade (D) and cluster (D1) was penicillin resistant.

REFERENCES TO S1.4. *Bacillus anthracis*

274. Inglesby, T. V., O'Toole, T., Henderson, D. A., Bartlett, J. G., Ascher, M. S., Eitzen, E., Friedlander, A. M., Gerberding, J., Hauer, J., Hughes, J., McDade, J., Osterholm, M. T., Parker, G., Perl, T. M., Russell, P. K. and Tonat, K. (2002). Anthrax as a biological weapon, 2002: updated recommendations for management. *JAMA* 287, 2236-2252. DOI: 10.1001/jama.287.17.2236.

275. Ruiz, J. (2003). Mechanisms of resistance to quinolones: target alterations, decreased accumulation and DNA gyrase protection. *J. Antimicrob. Chemother.* 51, 1109-1117. DOI: 10.1093/jac/dkg222.

276. Aldred, K. J., McPherson, S. A., Wang, P., Kerns, R. J., Graves, D. E., Turnbough, C. L. Jr. and Osheroff, N. (2012). Drug interactions with *Bacillus anthracis* topoisomerase IV: biochemical basis for quinolone action and resistance. *Biochem.* 51, 370-381. DOI: 10.1021/bi2013905.

277. Markham, P. N. and Neyfakh, A. A. (2001). Efflux-mediated drug resistance in Gram-positive bacteria. *Curr. Opin. Microbiol.* 4, 509-514. DOI: 10.1016/S1369-5274(00)00243-5.

278. Serizawa, M., Sekizuka, T., Okutani, A., Banno, S., Sata, T., Inoue, S. and Kuroda, M. (2010). Genomewide screening for novel genetic variations associated with ciprofloxacin resistance in *Bacillus anthracis*. *Antimicrob. Agents Chemother.* 54, 2787-2792. DOI: 10.1128/AAC.01405-09.

279. Raskó, D. A., Worsham, P. L., Abshire, T. G., Stanley, S. T. Bannan, J. D., Wilson, M. R., Langham, R. J., Decker, R. S., Jiang, L., Read, T. D., Phillippy, A. M., Salzberg, S. L., Pop, M., van Ert, M. N., Kenefic, L. J., Keim, P. S., Fraser-Liggett, C. M. and Ravel, J. (2011). *Bacillus anthracis* comparative genome analysis in support of the Amerithrax investigation. *Proc. Natl. Acad. Sci.* 108, 5027-5032. DOI: 10.1073/pnas.1016657108.

280. Kreizinger, Z., Sulyok, K. M., Makrai, L., Rónai, Z., Fodor, L., Jánosi, S. and Gyuranecz, M. (2016). Antimicrobial susceptibility of *Bacillus anthracis* strains from Hungary. *Acta Vet. Hung.* 64, 141-147. DOI: 10.1556/004.2016.015.

281. Pilo, P., Rossano, A., Bamamga, H., Abdoulkadiri, S., Perreten, V. and Frey, J. (2011). Bovine *Bacillus anthracis* in Cameroon. *Appl. Environ. Microbiol.* 77, 5818-5821. DOI: 10.1128/AEM.00074-11.

282. World Health Organization. (2007). WHO guidelines on tularaemia: epidemic and pandemic alert and response. World Health Organization.

SUPPLEMENTARY MATERIAL 2 / Fodor et al. 2020

RESISTANCE MECHANISMS ACQUIRED AND/OR PERFORMED BY ENTEROCOCCI

The entry '*Enterococci resistance mechanisms'* I infections' invokes altogether 6282 items (articles, including 3707 Reviews, which is an unusual ration.) from PubMed. This subject had recently been reviewed historically first by (Hidron et al., 2008) and most comprehensively by Miller et al., (Miller al., 2014).

It is known that the antibiotic resistance can be materialized by enzymatic detoxification, efflux, decreased cell wall permeability (that is decreased the affinity of the target for the antibiotic), and bypass of the target. For β-lactams, detoxification of the antibiotics by β-lactamases is widespread in nearly all bacterial phyla. In Gram-negative bacteria, β-lactamase production is frequently associated with reduced permeability of the outer membrane and efflux. However, in the Gram-positive ones, this permeability barrier does not exist, and resistance is often due to the production of targets displaying a lower affinity for the respective antibiotics (Sacco et al., 2015). The fourth bypass mechanism has been identified for the first time in an ampicillin mutant hunt experiment with *E. faecium* (Murray, 1992). In these mutants, the classical targets of β-lactams, the high-molecular-weight penicillin-binding proteins (PBPs) are replaced by an l, d-transpeptidase (LDT), which catalyzes the essential cross-linking step of peptidoglycan synthesis.

Resistance mechanisms to beta-lactams (penicillin and ampicillin)

A tricky mechanism of beta-lactam resistance due to bypass of dd-transpeptidation is performed by *Enterococcus faecium*, Mainardi (et al, 2000). The most important factor is that resistance toward cell-wall active antibiotics is not restricted to intrinsic lactamase activity (Ono et al., 2005), but several others factors (Duez et al., 2001) related to structural or expression-rate changes of penicillin-binding proteins: over-production low-affinity binding (Rice et al., 2004), or mutations affecting the structure or regulation of a penicillin-binding protein (Montealegre et al., 2017). Publications appearing since 2014 confirm differential penicillin-binding protein 5 (PBP5) levels in the *E. faecium* clades with different levels of ampicillin resistance (Zhang et al., 2012). The genome-wide identification of ampicillin resistance determinants in *E. faecium* revealed that although mutations in the low-affinity penicillin-binding protein PBP5 have played an important, but not exclusive, the role for ampicillin resistance in this species (Rice et al., 2009), (although the existence of additional resistance determinants has also been suggested, see, for instance https://www.ncbi.nlm.nih.gov/pmc/articles/PMC3386183/). The authors constructed a high-density transposon

mutant library for E. faecium and developed a transposon mutant tracking approach termed Microarray-based Transposon Mapping (M-TraM). This approach led to the identification of a compendium of E. faecium genes that contribute to ampicillin resistance. These genes are part of the core genome of E. faecium, indicating a high potential for it to evolve towards β-lactam resistance (Rice et al., 2009). Furthermore, they validated their M-TraM results by adopting a Cre-lox recombination system to construct targeted, marker-less, mutants. They confirmed the role of 4 more genes in ampicillin resistance by the generation of targeted mutants and further characterized these mutants regarding their resistance to lysozyme. They showed that ddcP, a gene predicted to encode a low-molecular-weight penicillin-binding protein with D-alanyl-D-alanine carboxypeptidase activity, was essential for high-level ampicillin resistance. Furthermore, deletion of ddcP sensitized *E. faecium* to lysozyme and abolished membrane-associated D, D-carboxypeptidase activity (Rice et al., 2009). We consider this study to be of historic importance, by allowing the building of a broadly applicable platform for functional genomic-based studies in *E. faecium*.

Resistance mechanisms of Enterococci to cephalosporins

This resistance mechanism can be achieved by reducing their binding capacities (Le Breton et al., 2007). This subject had also recently been thoroughly reviewed by Miller et al. (Hidron et al., 2008). In bacteria, several regulatory pathways controlled by bacterial two-component regulatory systems (TCS), like that of the CroRS two-component system in E. faecalis (Snyder et al., 2014), may also be associated with the intrinsic resistance to cephalosporins. Experimental proof of the existence of a nutritional control mechanism of antibiotic resistance mediated by the phosphotransferase system, and a two-component signaling system, has recently been published (Hall et al., 2013). A third protein, IreB, a Ser/Thr kinase substrate also plays a role (Djorić and Kristich, 2015). Since then, one publication came out confirming the role of an inducible, two-component, signaling system, in the cephalosporin resistance of *E. faecalis* (Courvalin, 2006).

Resistance mechanisms of Enterococci to glycopeptide antibiotics such as vancomycin and teicoplanin

The glycopeptide antibiotics vancomycin and teicoplanin bind to the peptidyl-D-Ala4-F-Ala5 extremity of peptidoglycan precursors, and cause inhibition by steric hindrance of the elongation of both glycan chains, by glycosyl-transferases, and the cross-linking of stem peptides by D, D-trans-peptidases (Murray, 1992). The L, D-trans-peptidases use acyl donors, and they contain a stem tetra-peptide ending in D-Ala4 that does not bind to these antibiotics. A novel peptidoglycan cross-linking enzyme changing the terminal amino acids of the peptidoglycan precursor from D-Ala-D-Ala to D-alanine-D-lactate (d-Ala-d-Lac) or, sometimes to D-alanine-D-serine (D-Ala-D-Ser) results in resistance to glycopeptide antibiotic. This and other possible resistance mechanisms were also considered (Novotna et al., 2012).

The literature related to the resistance mechanisms of enterococci to glycopeptide antibiotics had recently been reviewed thoroughly (Hidron et al., 2008). Since then, however, two important discoveries have been published. First, the novel membrane protein called VanJ is considered to confer resistance to teicoplanin (Takahiro et al., 2012) and vancomycin (Niu et al., 2016).

Second, a whole-genome sequence has been performed (Sacco et al., 2015) to identify the complete set of mutations occurring during selective pressure of elevated dose of antibiotics. This extremely impressive study, carried out by Sacco and his associates, is probably worth a little more detailed discussion. (It should be taught in student courses).

The authors produced a strain (called M9); containing 79 relevant mutations obtained through 9 selections steps from 900 generations. At the end of each selection step, they isolated a respective multi-mutant strain and named them M1–M9. The parental strain D344S M9 was completely sensitive, while M9 was fully resistant to ampicillin, vancomycin, and tetracycline. They found that the ddC locus was not affected through 4 selection steps, and mutant strains M1–M4 remained sensitive to each of the three antibiotics, although the resistance to ampicillin seemed to be gradually growing. The 5th selection step was critical, resulting in M512, fully resistant to ampicillin, but still sensitive to tetracycline, and showing a moderate vancomycin resistance. The ddC gene was inactivated. In the next 3 generations, the resistance towards vancomycin and tetracycline gradually grew, and M8 was fully resistant to vancomycin.

The whole genome sequencing procedure, comparing the parental (D344S) and the various mutant M9 strains was carried out by Illumina single read sequencing technology. The Illumina library preparation (genomic DNA sample prep kit v1) and sequencing followed standard protocols developed by the supplier. They found that the genome of mutant M9s differed from that of the parental strain E. faecium D344S by a total of 79 mutations. Sanger sequencing was performed to confirm the presence of the 79 mutations in M9, and to assign each of the mutations to one of the nine selection steps used to obtain mutant M9.

The authors found that among the mutations detected in M9, 65 were nonsynonymous mutations. Assignment of the corresponding proteins in functional classes revealed sequence alterations in eight proteins involved in transcription regulation, including CroR, a response regulator of a two-component regulatory system that contributes to intrinsic β-lactam resistance in the enterococci by an unknown mechanism. Nonsynonymous mutations also affected two sensor kinases, suggesting that regulatory circuits involving two-component regulatory systems are affected in response to the acquisition of ampicillin and glycopeptide resistance.

These results suggest that the fluxes through central metabolic pathways, including glycolysis, might be profoundly remodeled in a mutant set of M9. This is an indirect confirmation of the recent report on E. faecalis mutants hyper-susceptible to β-lactam antibiotics (Hall et al., 2013), indicating that existence of nutritional control of antibiotic resistance is based on a connection between CroR and the phosphotransferase system (PTS)system. As for the peptidoglycan synthesis, they found that 4 of the 20 enzymes, committed to peptidoglycan biosynthesis, were affected by amino substitutions. None of the substitutions had any obvious role in the activation of the L, D-transpeptidation pathway.

An important observation is that the substitutions did not involve enzymes that recognize the peptide stems of peptidoglycan precursors (Sacco et al., 2015).

Vancomycin-resistant enterococci (VRE) gene operons perform distinct genetic heterogeneity and spectacularly continued evolution resulting in a variety of antimicrobial resistance phenotypes, as concluded also by Ahmed and Baptiste (2018).

Resistance mechanisms to antibiotics that interfere with protein synthesis

Enterococci display intrinsic tolerance to aminoglycosides (Bender et al., 2016). Mutations in genes encoding the 23S rRNA, which is an important part of the drug-binding site at the ribosome, are the most common mechanisms for linezolid resistance (Hidron et al., 2008; Si et al., 2015).

The **streptogramins/macrolides/lincosamides** are a mixture of pristinamycin derivatives, streptogramin A (dalfopristin) and B quinupristin, which are effective against *E. faecium*, but not *E. faecalis*.

Lincosamide and streptogramin A resistance in *E. faecalis* has the respective chromosomally located resistance gene encoding for a putative protein with an ATP-binding cassette motif of transporter proteins, but not the transmembrane region that would be expected for an efflux pump (Hidron et al., 2008; Maasjost et al., 2015).

Cross-resistance with all macrolides are a result of the modification of the 23S rRNA target. Resistance to tetracyclines and glycylcyclinesis is mediated by multiple genes but follows two general strategies: efflux of the antibiotics and ribosomal protection. Mutations in the genes, gyrA and parC, (present in *E. faecium* and *E. faecalis*, but absent from *E. gallinarum* and *E. casseliflavus*) affect the quinolone resistance-determining regions, which presumably alter the binding affinity of the antibiotic quinolones, the target enzymes (DNA gyrase and topoisomerase IV) that are responsible for DNA supercoil relaxation (Hidron et al., 2008; Sacco et al., 2015). **Rifampicin resistance** arises from a variety of mutations in the rpoB gene that encodes for the β-subunit of the RNA polymerase (Hidron et al., 2008). **Trimethoprim and sulfamethoxazole** (inhibitors of bacterial enzymes involved in the folate synthesis pathway) are ineffective *in vivo* to enterococci because they are those extremely rare bacteria which can utilize exogenous sources of folate (Hidron et al., 2008; Sacco et al., 2015).

Resistance mechanisms to the lipo-protein antibiotics daptomycin

Daptomycin (DAP) is NRP lipopeptide consisting of a peptide core and a lipid tail with a diverse array of target organisms and modes of action with specific activity against Gram-positive organisms. DAP used

to be believed as the last trump-card against Gram/positive pathogens until the emergence of resistance to DAP. The mechanisms performed by Enterococci have recently been reviewed by Miller et al. (2015).

REFERENCES

1. Ahmed, M. O., Baptiste, K. E. (2018). Vancomycin-Resistant Enterococci: A Review of Antimicrobial Resistance Mechanisms and Perspectives of Human and Animal Health. *Microb. Drug Resist.* 5, 590-606. doi: 10.1089/mdr.2017.0147. Epub 2017 Oct 23. Review. PMID: 29058560.

2. Bender, J. K., Fleige, C., Klare, I., Fiedler, S., Mischnik, A., Mutters, N. T., Dingle, K. E. and Werner, G. (2016). Detection of a cfr(B) variant in German *Enterococcus faecium* clinical isolates and the impact on linezolid resistance in Enterococcus spp. *PLoS One* 11, e0167042. DOI: 10.1371/journal.pone.0167042.

3. Courvalin, P. (2006). Antibiotic resistance: the pros and cons of probiotics. *Dig. Liver Dis.* 38, 261-265. DOI: 10.1016/S1590-8658(07)60006-1.

4. Djorić, D. and Kristich, C. J. (2015). Oxidative stress enhances cephalosporin resistance of *Enterococcus faecalis* through activation of a two-component signaling system. *Antimicrob. Agents Chemother.* 59, 159-169. DOI: 10.1128/AAC.03984-14.

5. Duez, C., Zorzi, W., Sapunaric, F., Amoroso, A., Thamm, I. and Coyette, J. (2001). The penicillin resistance of *Enterococcus faecalis* JH2-2r results from an overproduction of the low-affinity penicillin-binding protein PBP4 and does not involve a psr-like gene. *Microbiol.* 147, 2561-2569. DOI: 0.1099/00221287-147-9-2561.

6. Hall, C. L., Tschannen, M., Worthey, E. A. and Kristich, C. J. (2013). IreB, a Ser/Thr kinase substrate, influences antimicrobial resistance in *Enterococcus faecalis*. *Antimicrob. Agents Chemother.* 57, 6179-6186. DOI: 10.1128/AAC.01472-13.

7. Hidron, A. I., Edwards, J. R., Patel, J., Horan, T. C., Sievert, D. M., Pollock, D. A. and Fridkin, S. K. (2008). Antimicrobial-resistant pathogens associated with healthcare-associated infections: annual summary of data reported to the National Healthcare Safety Network at the Centers for Disease Control and Prevention, 2006-2007. *Infect. Control Hosp. Epidemiol.* 29, 996-1011. DOI: 10.1086/59186

8. Le Breton, Y., Muller, C., Auffray, Y. and Rincé, A. (2007). New insights into the *Enterococcus faecalis* CroRS two-component system obtained using a differential-display random arbitrarily primed PCR approach. *Appl. Environ. Microbiol.* 73, 3738-3741. DOI: 10.1128/AEM.00390-07.

9. Maasjost, J., Mühldorfer, K., Cortez de Jäckel, S. and Hafez, H. M. (2015). Antimicrobial susceptibility patterns of *Enterococcus faecalis* and *Enterococcus faecium* isolated from

poultry flocks in Germany. *Avian Dis.* 59, 143-148. DOI: 10.1637/10928-090314-RegR.

10. Mainardi, J. L., Legrand, R., Arthur, M., Schoot, B., van Heijenoort, J. and Gutmann, L. (2000). Novel mechanism of beta-lactam resistance due to bypass of dd-transpeptidation in *Enterococcus faecium*. *J. Biol. Chem.* 275, 16490-16496. DOI: 10.1074/jbc.M909877199.

11. Miller, W. R., Bayer, A. S., Arias, C. A. (2015). Mechanism of Action and Resistance to Daptomycin in *Staphylococcus aureus* and Enterococci. *Cold Spring Harb. Perspect. Med.* 6, 11. pii: a026997. doi: 10.1101/cshperspect.a026997.

12. Miller, W. R., Munita, J. M. and Arias, C. A. (2014). Mechanisms of antibiotic resistance in Enterococci. *Expert Rev. Anti. Infect. Ther.* 12, 1221-1236. DOI: 10.1586/14787210.2014.956092./

13. Montealegre, M. C., Roh, J. H., Rae, M., Davlieva, M. G., Singh, K. V., Shamoo, Y. and Murray, B. E. (2017). Differential penicillin-binding protein 5 (PBP5) Levels in the *Enterococcus faecium* clades with different levels of ampicillin resistance. *Antimicrob. Agents Chemother.* 61, e02034-16. DOI: 10.1128/AAC.02034-16.

14. Murray, B. E. (1992). Beta-lactamase-producing Enterococci. *Antimicrob. Agents Chemother.* 36, 2355-2359.

15. Niu, H., Yu, H., Hu, T., Tian, G., Zhang, L., Guo, X., Hu, H. and Wang, Z. (2016). The prevalence of aminoglycoside-modifying enzyme and virulence genes among Enterococci with high-level aminoglycoside resistance in Inner Mongolia, China. *J. Microbiol.* 47, 691-696. DOI: 10.1016/j.bjm.2016.04.003.

16. Novotna, G., Hill, C., Vincent, K., Liu, C. and Hong, H. J. (2012). A Novel membrane protein, VanJ, conferring resistance to teicoplanin. *Antimicrob. Agents Chemother.* 56, 1784-1796. DOI: 10.1128/AAC.05869-11\

17. Ono, S., Muratani, T. and Matsumoto, T. (2005). Mechanisms of resistance to imipenem and ampicillin in *Enterococcus faecalis*. *Antimicrob. Agents Chemother.* 49, 2954-2958. DOI: 10.1128/AAC.49.7.2954-2958.2005.

18. Rice, L. B., Bellais, S., Carias, L. L., Hutton-Thomas, R., Bonomo, R. A., Caspers, P., Page, M. G. and Gutmann, L. (2004). Impact of specific pbp5 mutations on expression of beta-lactam resistance in *Enterococcus faecium*. *Antimicrob. Agents Chemother.* 48, 3028-3032. DOI: 10.1128/AAC.48.8.3028-3032.2004.

19. Rice, L. B., Carias, L. L., Rudin, S., Hutton, R., Marshall, S., Hassan, M., Josseaume, N., Dubost, L., Marie, A. and Arthur, M. (2009). Role of class a penicillin-binding protein in the expression of beta-lactam resistance in *Enterococcus faecium*. *J. Bacteriol.* 191, 3649-3656. DOI: 10.1128/JB.01834-08.

20. Sacco, E., Cortes, M., Josseaume, N., Bouchier, C., Dubée, V., Hugonnet, J. -E., Mainardi, J. L., Rice, L. B. and Arthur, M. (2015). Mutation landscape of acquired cross-resistance to glycopeptide and β-lactam antibiotics in *Enterococcus faecium*. *Antimicrob. Agents Chemother.* 59, 5306-5315. DOI: 10.1128/AAC.00634-15.

21. Snyder, H., Kellogg, S. L., Skarda, L. M., Little, J. L. and Kristich, C. J. (2014). Nutritional control of antibiotic resistance via an interface between the phosphotransferase system and a two-component signaling system. *Antimicrob. Agents Chemother.* 58, 957-965. DOI: 10.1128/AAC.01919-13.

22. Takahiro, N., Koichi, T., Keigo, S., Yoshichika, A., Shuhei, F., Yasuyoshi, I. and Haruyoshi, T. (2012). Identification of VanN-Type vancomycin resistance in an *Enterococcus faecium* Isolate from chicken meat in Japan. *Antimicrob. Agents Chemother.* 56, 6389-6392. DOI: 10.1128/AAC.00747-12.

23. Si, H., Zhang, W. J., Chu, S., Wang, X. M., Dai, L., Hua, X., Dong, Z., Schwarz, S. and Liu, S. (2015). Novel plasmid-borne multidrug resistance gene cluster including lsa(E) from a linezolid-resistant *Enterococcus faecium* isolate of swine origin. *Antimicrob. Agents Chemother.* 59, 7113-7116. DOI: 10.1128/AAC.01394-15.

24. Zhang, X., Paganelli, F. L., Bierschenk, D., Kuipers, A., Bonten, M. J., Willems, R. J. and van Schaik, W. (2012). Genome-wide identification of ampicillin resistance determinants in *Enterococcus faecium*. *PLoS Genet.* 8, e1002804. DOI: 10.1371/journal.pgen.1002804.

SUPPLEMENTARY MATERIAL 3 / Fodor et al

The Speed of Development of Antibiotic Multi-Drug Resistance in Genus *Enterococcus* is Rather a Revolutionary than Evolutionary

Enterococcus cecorum, a normal commensal intestinal inhabitant is increasingly responsible for out-breaks of arthritis and osteomyelitis in chickens worldwide. However, since 2002, *E. cecorum* has increasingly been recognized as the causative pathogen of enterococcal spondylitis (ES), (Devriese et al., 2002; Gilmore, 2002; Wood et al., 2002; Chadfield et al., 2004; Debnam et al., 2005; Thayer et al., 2008; Aziz and Barnes, 2007; Aziz and Barnes, 2009; DeHerdt et al., 2008; Gingerich, 2009; Stalker et al., 2010; Martin et al., 2011; Boerlin et al., 2012).

Enterococcal spondylitis is a specific manifestation of *E. cecorum*-associated diseases, in which increased flock morbidity and mortality resulting from a chronic infection involving the free thoracic vertebra and adjacent notarium or synsacrum. Birds affected with ES have hind-limb paresis of variable severity, due to spinal cord compression caused by the chronic inflammation, (DeHerdt et al., 2008; Aziz and Barnes, 2009; Gingerich, 2009; Stalker et al., 2010). To date, ES has been reported in both breeding and meat production flocks in several U.S. states, (Aziz and Barnes, 2009; Gingerich, 2009). ES has also been documented in broiler chickens in different countries all over the globe, in Belgium (Devriese et al., 2002) in Canada (Wood et al., 2002), in Hungary (Makrai et al., 2011), in Poland, (Dolka et al., 2016), in The Netherlands, (Boerlin et al., 2012), and the UK, (Kense and Landman, 2011). Clinical presentations, gross findings and epidemiology are like those found in American broiler flocks (Gingerich, 2009). Although the pathogenesis of ES remains is poorly understood, recent evidence suggests that the increased incidence of enterococcal-associated disease in poultry maybe due to the horizontal spread of dominant clones of *E. cecorum* that exhibit increased pathogenicity (Boerlin et al., 2012; Kense and Landman, 2011. Antimicrobial resistance of isolates recovered from spondylitis lesions and caeca of affected/ unaffected birds from geographically and temporally distinct outbreaks of ES in the southeastern United States (Boerlin et al., 2012). Data indicate that the increase in *E. cecorum*-associated diseases in the the southeast United States is due to the emergence of new clones with increased pathogenicity and multi-drug resistance, (Borst et al., 2012).

REFERENCES

Aziz, T. and Barnes, H. J. (2007). Is spondylitis an emerging disease in broilers? *World Poultry* 23, 44-45.

Aziz, T. and Barnes, H. J. (2009). Spondylitis is emerging in broilers. *World Poultry* 25,19.

Boerlin, P., Nicholson, V., Brash, M., Slavic, D., Boyen, F., Sanei, B. and Butaye, P. (2012). Diversity of *Enterococcus cecorum* from chickens. *Vet. Microbiol.* 157, 405-411. DOI: 10.1016/j.vetmic.2012.01.001

Borst, L. B., Suyemoto, M. M., Robbins, K. M., Lyman, R. L. Martin, M. P. and Barnes, J. H. (2012). Molecular epidemiology of *Enterococcus cecorum* isolates recovered from Enterococcal spondylitis outbreaks in the southeastern United States. *J. Pathol.* 41, 479-485. DOI: 10.1080/03079457.2012.718070

Chadfield, M. S., Christensen, J. P., Christensen, H. and Bisgaard, M. (2004). Characterization of Streptococci and Enterococci associated with septicaemia in broiler parents with a high prevalence of endocarditis. *Avian Pathol.* 33, 610-617. DOI: 10.1080/03079450400013089.

Debnam, A. L., Jackson, C. R., Avellaneda, G. E., Barrett, J. B. and Hofacre, C. L. (2005). Effect of growth promotant usage on Enterococci species on a poultry farm. *Avian Dis.* 49, 361-365. DOI: 10.1637/7328-011405R.1.

DeHerdt, P., Defoort, P., Steelant, J. V., Swam, H., Tanghe, L., Goethem, S. V. and Vanrobaeys, M. (2008). *Enterococcus cecorum* osteomyelitis and arthritis in broiler chickens. *Vlaams Diergeneeskundig Tijdschrift* 78, 44-48.

Devriese, L. A., Cauwerts, K., Hermans, K. and Wood, A. M. (2002), Vlaams Diergeneeskundig Tijdschrif 71, 219.

Dolka, B., Chrobak-Chmie, D., Makrai, L. and Szeleszczuk, P. (2016). Phenotypic and genotypic characterization of *Enterococcus cecorum* strains associated with infections in poultry. *BMC Vet. Res.* 12, 129. DOI: 10.1186/s12917-016-0761-1.

Gilmore, M. 2002, The Enterococci: Pathogenesis, molecular biology and antimicrobial resistance. Washington, DC, *ASM Press.*

Gingerich, E. (2009). Emerging disease syndrome: spinal abscesses hit US broiler flocks. *WATT Poultry USA* 10, 24-32.

Kense, M. J. and Landman, W. J. M. (2011). *Enterococcus cecorum* infections in broiler breeders and their offspring: molecular epidemiology. *Avian Pathol.* 40, 603-612. DOI: 10.1080/03079457.2011.619165.

Makrai, L., Nemes, C., Simon, A., Ivanics, E., Dudás, Z., Fodor, L. and Glavits, R. (2011). Association of *Enterococcus cecorum* with vertebral osteomyelitis and spondylolisthesis in broiler parent chicks. *Acta Vet. Hung.* 59, 11.

Martin, L. T., Martin, M. P. and Barnes, H. J. (2011). Experimental reproduction of Enterococcal spondylitis in male broiler breeder chickens. *Avian Dis.* 55, 273-278. DOI: 10.1637/9614-121410-Reg.1.

Stalker, M. J., Brash, M. L., Weisz, A., Ouckama, R. M. and Slavic, D. (2010). Arthritis and osteomyelitis associated with *Enterococcus cecorum* infection in broiler and broiler breeder chickens in Ontario, Canada. *J. Vet. Diag. Invest*. 22, 643-645. DOI: 10.1177/104063871002200426.

Thayer, S. G., Waltman, W. D. and Wages, D. P. (2008). Streptococcus and Enterococcus. *Diseases of Poultry* 12, 900-908.

Wood, A. M., MacKenzie, G., McGillveray, N. C., Brown, L., Devriese, L. A. and Baele, M. (2002). Isolation of *Enterococcus cecorum* from bone lesions in broiler chickens. *Vet. Record.* 150, 27.

SUPPLEMENTARY MATERIAL 4 / Fodor et al

Dialectics of Resistance and Sensitivity in The Genus *Agrobacterium*

(The agrocin 84 story).

So far, we have discussed resistance genes, and resistance mutations, the existence, and expression of which make the originally sensitive organism resistant to a given compound. The product of the mutant gene causing resistance can be located either on the chromosome, or on a newly taken-up plasmid, harboring it either as part of a mobile genetic element, or genomic island. But there are examples of the opposite as well when the existence and expression of a "sensitivity gene" make the originally resistant organism sensitive to a given antibacterial compound.

The protein product of such a sensitivity gene may catabolize a harmless molecule to a harmful derivative or change the structure of the originally impermeable membrane to be permeable to the compound, or to block the multi-drug pumping activity. Alternatively, a sensitivity gene may code for a protein which can bind and transfer a toxic product into the cell, which it could not otherwise enter. This binding protein may play a role in the normal metabolism of the given cell. A wonderful example is a sensitivity/resistance (S/R) phenotype in different Agrobacterium strains to Agrocin 84. It is one of the most studied molecules of the group of plant biocontrol molecules called agrocins [330] (Clare, 1995). This "Trojan horse antibiotic" that controls the plant tumor called crown gall (Kim et al., 2006) is an adenine nucleotide antibiotic, produced by and discovered in, an avirulent Biovar 2 strain of *A. radiobacter* 84.

Three *Agrobacterium* species have been identified so far: *A. tumefaciens*, also called Biovar I; *A. radiobacter*, Biovar 2; and *A. rhizogenes*, Biovar 3 [(Wood et al., 2001). Each species includes virulent and avirulent strains. The virulent strains induce specific tumors, characterized by their secondary metabolites called opines. Depending on the type of opines, the virulent Agrobacterium strains of each species could be determined to be in the nopaline (NO), octopine (OCT), or agropine (AGR) opine group. Each Agrobacterium strain can induce a special opine-synthetizing tumor, and each of them can catabolize the respective opine, (although the respective opine was synthesized by the tumorous plant cells and not the bacterium). Agrocin 84 was first identified as trypsin and a pepsin-resistant small peptide with a molecular weight of 2,500. It was published as being built up of six different amino acids, including 9 molecules of glutamine or glutamic acid, and seven molecules of serine. It inhibited DNA, RNA and protein synthesis as well as amino acid transport of the virulent, susceptible, A. tumefaciens (H38-9) strain (McCardell et al., 1976). Agrocin 84 is toxic to several other but not all, *Agrobacterium* strains. Agrocin 84 inhibits those virulent, tumor-causing, Agrobacterium strains (called NOP strains), which induce nopaline-synthetizing tumor cells. The respective tumor-inducing Agrobacterium strains carry nopaline catabolizing genes on their respective (Ti), or hairy-root inducing (Ri), plasmid. If the plasmid carrying nopaline catabolizing genes were removed (cured) from the Agrocin 84-sensitive NOP strains, they became resistant to Agrocin 84. This phenomenon explains that the agrocinopine gene, which has a normal metabolic function in the nopaline biosynthesis in the wild-type strain, also pleiotropically functions as a "sensitivity" gene, making the wild-type NOP *Agrobacterium* strains sensitive to Agrocin 84.

For a better understanding, it is important to know that *A. tumefaciens* NOP strains, such as strain C58 (van Larebeke et al., 1974), induced crown gall tumors to produce not only nopaline but other opines, called agrocinopines A and B, as well. Agrocinopine A has a normal function

in NOP strains, where it is the inducer of Ti plasmid conjugal transfer in the strain (Ellis et al., 1982). It turned out that Agrocin 84 and agrocinopines A, the precursor of agrocinopine B, are transported by the same uptake system.

Consequently, mutations causing a constitutive transfer of pTiC58 show the pleiotropic phenotype of super-sensitivity to Agrocin 84, while Agrocin 84-resistant mutants of A. tumefaciens A208 do not transport agrocinopine A. Other *Agrobacterium* strains harboring non-NOP, pTiBo542 plasmids (which induce L, L, succinamopine, and agropine producing tumors), the cells which also synthesize agrocinopines, but of a different type (agrocinopines C, the precursor of agrocinopine D), are resistant to Agrocin 84, but could be made sensitive by pretreatment with agrocinopine C, (Clare, 1995). For more details, see (Bomhoff et al., 1976; Murphy and Roberts, 1979; Ellis et al., 1979; Ellis and Murphy, 1981; Ellis et al., 1982; Petit et al., 1982; Ryder et al., 1984; Farrand et al., 1985; Hayman and Farrand, 1988; Hayman and Farrand, 1990).

The authors of this review have a reservation for accepting this pessimistic view.

We suppose that the “card game” between new antibiotics and invoked resistances has not been finished yet. The genetic sources of both intrinsic and acquired resistance in the bacteria seem to be non-exhaustible. This fact justifies ‘hands-up’ pessimism.

Fortunately, enough, the number of theoretically possible QSAR-designed antimicrobial peptides also seems to be unlimited, or at least extremely high. This fact justifies rational optimism, hoping that there is a real chance to overcome newly appearing resistances by discovering and introducing new, properly designed antimicrobial peptides at least for a long time.

Therefore, we are expecting new antimicrobial peptides (either of natural or of synthetic origin) as new trumps as in the “card game” of science and bacterial multi-drug resistance. In the “card game” of the antibiotics and invoked resistances many reliable investigators consider polymyxin (colistin) and vancomycin as the respective last “trump” against Gram-negative and Gram-positive resistant pathogens, forecasting that the appearance Gram-negative isolates of colistin-resistance and that of Gram-positive isolates of vancomycin resistance means the end of the heroic “age of antibiotics”.

We propose that the options provided by the natural and synthetic antimicrobial peptides will offer new solutions. The QSAR-designed synthetic antimicrobial peptides and the non-ribosomal (NRP) peptides, especially those produced by entomopathogenic nematode symbiotic bacteria (*Xenorhabdus, Photorhabdus*) provide abundant gold mines for antibiotics of novel modes of action (see our next Review).

As a very personal epilog, let us explain why we, practically newcomers, did undertake to put this review together. Our team has been working on natural antimicrobial peptides produced by entomopathogenic nematode symbiotic bacteria and have recently started to cooperate with a team of veterinary scientists to materialize this conception. This review is our first joint venture.

REFERENCES

Clare, B. G. (1995). Genetics and Biochemistry of Antibiotic Production. 619

Kim, J. G., Park, B. K., Kim, S. U., Choi, D., Nahm, B. H., Moon, J. S., Reader, J. S., Farrand, S. K. and Hwang, I. (2006). Bases of biocontrol: sequence predicts synthesis and mode of action of agrocin 84, the Trojan horse antibiotic that controls crown gall. *Proc.Natl. Acad. Sci.* 103, 8846-8851. DOI: 10.1073/pnas.0602965103.

Wood, D. W., Setubal, J. C., Kaul, R., Monks, D. E., Okura, V. K., Zhou, Y., Chen, L., Wood, G. E., Almeida, N. F. Jr., Woo, L., Chen, Y., Paulsen, I. T., Eisen, J. A., Karp, P. D., Bovee, D. Sr., Chapman, P., Clendenning, J., Deatherage, G., Gillet, W., Grant, C., Kutyavin, T., Levy, R., Li, M. J., McClelland, E., Palmieri, A., Raymond, C., Rouse, G., Saenphimmachak, C., Wu, Z., Romero, P., Gordon, D., Zhang, S., Yoo, H., Tao, Y., Biddle, P., Jung, M., Krespan, W., Perry, M., Gordon- Kamm, B., Liao, L., Kim, S., Hendrick, C., Zhao, Z. Y., Dolan, M., Chumley, F., Tingey, S. V., Tomb, J. F., Gordon, M. P., Olson, M. V. and Nester, E. W. (2001). The genome of the natural genetic engineer *Agrobacterium tumefaciens* C58. *Science* 294, 2317-2323. DOI: 10.1126/science.1066804.

McCardell, B. A. and Pootjes, C. F. (1976). Chemical nature of Agrocin 84 and its effect on a virulent strain of *Agrobacterium tumefaciens*. *Antimicrob. Agents Chemother.* 10, 498-502. DOI: 10.1128/AAC.10.3.498.

van Larebeke, N., Engler, G., Holsters M., van den Elsacker, S., Zaenen, I., Schilperoort, R. A. and Schell, J. (1974). Large plasmid in *Agrobacterium tumefaciens* essential for crown gall-inducing ability. *Nature* 252, 169-170.

Ellis, J. G., Kerr, A., Petit, A. and Tempe, J. (1982). Conjugal transfer of nopaline and agropine Ti-plasmids-the role of agrocinopines. *Mol. Gen. Genet.* 186, 269-274.

Bomhoff, G., Klapwijk, P. M., Kester, H.C.M., Shilperoort, R. A., Hernalsteens, J. P. and Schell, J. (1976). Octopine and nopaline synthesis and breakdown genetically controlled by a plasmid of *Agrobacterium tumefaciens*. *Mol. Gen. Genet.* 145, 177-181.

Murphy, P. J. and Roberts, W. P. (1979). A Basis for Agrocin 84 sensitivity in *Agrobacterium radiobacter*. *J. Gen. Microbiol*. 114, 207-213. DOI: 10.1099/00221287-114-1-207.

Ellis, J. G., Kerr, A., Van Montagu, M. and Schell, J. (1979). Agrobacterium genetic studies on agrocin 84 production and the biological control of crown gall. *Physiol. Plant Pathol.* 15, 311-319. DOI: 10.1016/0048-4059(79)90082-1.

Ellis, J. G. and Murphy, P. J. (1981). Four new opines from crown gall tumors-their detection and properties. *Mol. Gen. Genet*. 181, 36-43.

Ellis, J. G., Murphy, P.J., and Kerr, A. (1982). Isolation and properties of transfer regulatory mutants of the nopaline Ti plasmid pTiC58. *Mol. Gen. Genet*. 186, 275-281.

Petit, A., David, C., Dahl, G. A., Ellis, J. G., Guyon, P., Casse-Delbart, F. and Tempe, J. (1982). Further extension of the opine concept: Plasmids in *Agrobacterium rhizogenes* cooperate for opine degradation. *Mol. Gen. Genet*. 190, 204-214.

Ryder, M. H., Tate, M. E. and Jones, G. P. (1984). Agrocinopine A, a tumor-inducing plasmid-coded enzyme product, is a phosphodiester of sucrose and L-arabinose. *J. Biol. Chem*. 259, 9704-9710.

Farrand, S. K., Slota, J. E., Shim, J. -S. and Kerr, A. (1985). Tn5 insertions in the agrocin 84 plasmid: The conjugal nature of pAgK84 and the locations of determinants for transfer and agrocin 84 production. *Plasmid* 13, 106-117. DOI: 10.1016/0147-619X(85)90063-0.

Hayman, T. G. and Farrand, S. K. (1988). Characterization and mapping of the Agrocinopine-Agrocin 84 locus on the nopaline Ti plasmid pTiC58. *J. Bateriol*. 170, 1759-1767. DOI: 10.1128/jb.170.4.1759-1767.1988.

Hayman, T. G. and Farrand, S. K. (1990). Agrobacterium plasmids encode structurally and functionally different loci for catabolism of agrocinopine-type opines. *Mol. Gen. Genet*. 223, 465-473.

Supplementary Text Material 5 *Pseudomonas aeruginosa* Antibiotic Resistances

Diseases: *P. aeruginosa* as an invasive the pathogen causes a wide range of diseases. As Mulcahy and his associates summarized, *P. aeruginosa* strains in human clinical practice are especially endangering immune-system-depressed or deficient patients, causing serious, nosocomial, human infections of the lower respiratory tract, the urinary tract; wounds in children and elder patients in hospitals, causing host-specific pathogenesis, (Mulcahy et al., 2010). The fatal consequences are most apparent in cystic fibrosis patients, where a high degree of biofilm formation was detected (Vahdani et al., 2012; Mulcahy et al., 2014). *P. aeruginosa* has also been a causative agent of infections in burn wounds (Peleg and Hooper, 2010), chronic wounds, a chronic obstructive pulmonary disorder, surface growth on implanted biomaterials, on hospital surfaces (Breathnach et al., 2012), and in water supplies (Gonçalves-de-Albuquerque et al., 2016), where it poses a host of threats to vulnerable.

Pathomechanisms: The first genome-wide search for and identification of genetic determinants of antimicrobial drug resistance in *Pseudomonas* was carried out by Dötch et al., (2009). Extracellular signals are capable of synchronizing a group of behaviors via gene regulation through a process called quorum sensing (QS), (Dötsch et al., 2012), making *P. aeruginosa* capable of forming massive biofilms, (Poole, 2001; Jeannot et al., 2008; Vahdani et al., 2012; Taylor et al., 2014; Guła et al., 2019). In the whole pan-genomic *P. aeruginosa* a complex QS system controls the expression of more than 300 genes (Dötsch et al., 2012; Chugani, and Grenberg, 2014), including many involved in host colonization and disease, (Yang and Lan, 2016; Rezaie et al., 2018

sense bacterial population density and fitness, to regulate virulence, biofilm development, metabolite acquisition, and mammalian host defense, (Turkina and Vikström, 2019). The regulatory and metabolic networks for the adaptation of *P. aeruginosa* biofilms to urinary tract-like conditions have been revealed, (Tielen et al., 2013).

References

Breathnach, A. S., Cubbon, M. D., Karunaharan, R. N., Pope, C. F. and Planche, T. D. (2012). Multidrug-resistant *Pseudomonas aeruginosa* outbreaks in two hospitals: association with contaminated hospital waste-water systems. *J. Hosp. Infect*. 82, 19-24. DOI: 10.1016/j.jhin.2012.06.007

Chugani, S. and Greenberg, E. P. (2014). An evolving perspective on the *Pseudomonas aeruginosa* orphan quorum sensing regulator QscR. Front. *Cell Infect. Microbiol.* 4, 152. DOI: 10.3389/fcimb.2014.00152

Dötsch, A., Becker, T., Pommerenke, C., Magnowska, Z., Jansch, L. and Haussler, S. (2009). Genome-wide identification of genetic determinants of antimicrobial drug resistance in *Pseudomonas aeruginosa*. *Antimicrob. Agents Chemother.* 53, 2522-2531. DOI: 10.1128/AAC.00035-09

Dötsch, A., Eckweiler, D., Schniederjans, M., Zimmermann, A., Jensen, V., et al., (2012). The *Pseudomonas aeruginosa* transcriptome in planktonic cultures and static biofilms using RNA sequencing. *PLoS One* 7, e31092. DOI: 10.1371/journal.pone.0031092

Gonçalves-de-Albuquerque, C. F., Silva, A. R., Burth, P., Rocco, P. R., Castro-Faria, M. V. et al., (2016). Possible mechanisms of *Pseudomonas aeruginosa*-associated lung disease. *Int. J. Med. Microbiol*. 306, 20-28. DOI: 10.1016/j.ijmm.2015.11.001

Guła, G., Dorotkiewicz-Jach, A., Korzekwa, K., Valvano, M. A. and Drulis-Kawa, Z. (2019). Complex signaling networks controlling dynamic molecular changes in *Pseudomonas aeruginosa* biofilm. *Curr. Med. Chem*. 26, 1979-1993. DOI: 10.2174/0929867325666180912110151

Jeannot, K., Elsen, S., Köhler, T., Attree, I., van Delden, C. and Plésiat, P. (2008). Resistance and virulence of *Pseudomonas aeruginosa* clinical strains overproducing the MexCD-OprJ efflux pump. *Antimicrob. Agents Chemother*. 52, 2455-2462. DOI: 10.1128/AAC.01107-07

Mulani, M. S., Kamble, E. E., Kumkar, S. N., Tawre, M. S. and Pardesi, K. R. (2019). Emerging strategies to combat ESKAPE pathogens in the era of antimicrobial resistance: A Review. *Front. Microbiol*. 10, 539. DOI: 10.3389/fmicb.2019.00539

Mulcahy, L. R., Burns, J. L., Lory, S. and Lewis, K. (2010). Emergence of *Pseudomonas aeruginosa* strains producing high levels of persister cells in patients with cystic fibrosis. *J. Bacteriol*. 192, 6191-6199. DOI: 10.1128/JB.01651-09

Mulcahy, L. R., Isabella, V. M. and Lewis, K. (2014). *Pseudomonas aeruginosa* biofilms in disease. *Microb. Ecol.* 68, 1-12. DOI: 10.1007/s00248-013-0297-x

Peleg, A. Y. and Hooper, D. C. (2010). Hospital-acquired infections due to gram-negative bacteria. *N. Engl. J. Med*. 362, 1804-1813. DOI: 10.1056/NEJMra0904124

Poole, K. (2001). Multidrug efflux pumps and antimicrobial resistance in *Pseudomonas aeruginosa* and related organisms. *J. Mol. Microbiol. Biotechnol.* 3, 255-264.

Rezaie, P., Pourhajibagher, M., Chiniforush, N., Hosseini, N. and Bahador, A. (2018). The effect of quorum-sensing and efflux pumps interactions in *Pseudomonas aeruginosa* against photooxidative stress. *J. Lasers Med. Sci.* 9, 161-167. DOI: 10.15171/jlms.2018.30

Taylor, P. K., Yeung, A. T. and Hancock, R. E. (2014). Antibiotic resistance in *Pseudomonas aeruginosa* biofilms: towards the development of novel anti-biofilm therapies. *J. Biotechnol*. 191, 121-130. DOI: 10.1016/j.jbiotec.2014.09.003

Tielen, P., Rosin, N., Meyer, A. -N., Dohnt, K., Haddad, I., et al., (2013). Regulatory and metabolic networks for the adaptation of *Pseudomonas aeruginosa* biofilms to urinary tract-like conditions. *PLoS One*. 8, e71845. DOI: 10.1371/journal.pone.0071845

Turkina, M.V. and Vikström, E. (2019). Bacteria-Host Crosstalk: Sensing of the Quorum in the Context of *Pseudomonas aeruginosa* Infections. *J. Innate Immun.* 11, 263-279. doi: 10.1159/000494069. Epub 2018 Nov 14. PMID: 30428481

Vahdani, M., Azimi, L., Asghari, B., Bazmi, F. and Rastegar Lari, A. (2012). Phenotypic screening of extended-spectrum ß-lactamase and metallo-ß-lactamase in multidrug-resistant *Pseudomonas aeruginosa* from infected burns. *Annal. Burns Fire Disast*. 25, 78-81.

Yang, N. and Lan, L. (2016). *Pseudomonas aeruginosa* Lon and ClpXP proteases: roles in linking carbon catabolite repression system with quorum-sensing system. *Curr. Genet.* 62, 1-6. DOI: 10.1007/s00294-015-0499-5

A shortlist of intrinsic and acquired antibiotic resistance in *P. aeruginosa*

Two recent excellent overview perfectly (Subedi et al., 2018; Mulani et al., 2019) provides "an ocular perspective" of the different mechanisms of antibiotic resistance in Pseudomonas aeruginosa. Most strains are resistant to the third generation cephalosporins produce a chromosomal-encoded C beta-lactamase and the AmpC enzymes (Nordmann et al., 1993); Lupo et al., 2018). The original intrinsic MDR-arsenal includes the production of beta-lactamases, loss of outer membrane proteins (Dhar et al., 2018) and up-regulation of efflux pumps, (Aeschlimann, 2003; Vila and Martínez, (2008); Fischer et al., 2014; Puzari and Chetia, 2017). This species also acquired resistance to aminoglycosides and fluoroquinolones, (Kidd et al., 2018; Pachori et al., 2019). One of these acquired enzymes taken-up by *P. aeruginosa*, (PER, *Pseudomonas extended resistance*) (Vahaboglu et al., 200; Szabó et al., 2008; Zhanel et al., 2019), a class of extended-spectrum beta-lactamase (ESBL) occurs less frequently, but still is of clinical importance (Queenan and Bush, 2007; Wozniak et al. 2019).

P. aeruginosa uses different mechanisms that can jointly contribute to its multi-resistant phenotype (Hirakata et al., 2002) and multi-drug efflux systems, (Nehme et al., 2004; Nehme and Poole, 2005; Jeukens et al., 2014; Jeukens et al., 2017; Jeukens et al., 2019). All of this makes *P. aeruginosa* extremely invasive. The rapidly increasing number of new *P. aeruginosa* isolates of MDR, XDR and PDR phenotypes, severely reduce the antibiotic therapy options available (El Zowalaty et al., 2015). As for the colistin, (acting on the outer membrane of the bacteria resulting in its permeability and cell-death) was suggested 5 years ago as salvage therapy in the treatment of often life-threatening infections due to MDR *P. aeruginosa* blood-stream infections (BSI), (Martis et al., 2014). Articles and reviews on resistance problems appeared one year later, (Catry et al., 2015; and appearing since then, Bialvaei et al., 2015, Potron et al,2015; Potron et al., 2019; (Romano et al, 2019).

References

Aeschlimann, J. R. (2003). The role of multidrug efflux pumps in the antibiotic resistance of *Pseudomonas aeruginosa* and other gram-negative bacteria: Insights from the Society of Infectious Diseases Pharmacists. *Pharmacotherapy*: *J. Human Pharmac. Drug Therapy* 23, 916-924. DOI: 10.1592/phco.23.7.916.32722

Bialvaei, A. Z. and Samadi Kafil, H. (2015). Colistin, mechanisms and prevalence of resistance. *Curr. Med. Res. Opin*. 31, 707-21. DOI: 10.1185/03007995.2015.1018989.

Catry, B., Cavaleri, M., Baptiste, K., Grave, K., Grein, K., et al., (2015). Use of colistin-containing products within the European Union and European Economic Area (EU/EEA): development of resistance in animals and possible impact on human and animal health. *Int J Antimicrob Agents.* 46, 297-306. doi: 10.1016/j.ijantimicag.2015.06.005. Epub 2015 Jun 29. Review. PMID: 26215780

Dhar, S., Kumari, H., Balasubramanian, D. and Mathee, K. (2018). Cell-wall recycling and synthesis in *Escherichia coli* and *Pseudomonas aeruginosa* - their role in the development of resistance. *J. Med. Microbiol*. 67, 1-21. DOI: 10.1099/jmm.0.000636

El Zowalaty, M. E., Al Thani, A. A., Webster, T. J., El Zowalaty, A. E., Schweizer, H. P., Nasrallah, G. K., et al.,. (2015). *Pseudomonas aeruginosa*: arsenal of resistance mechanisms, decades of changing resistance profiles, and future antimicrobial therapies. *Future Microbiol*. 10, 1683-1706. DOI: 10.2217/fmb.15.48

Fischer, N., Raunest, M., Schmidt, T. H., Koch, D. C. and Kandt, C. (2014). Efflux pump-mediated antibiotics resistance: insights from computational structural biology. *Interdiscip. Sci*. 6, 1-12. DOI: 10.1007/s12539-014-0191-3

Hirakata, Y., Srikumar, R., Poole, K., Gotoh, N., Suematsu, T., et al., (2002). Multidrug efflux systems play an important role in the invasiveness of *Pseudomonas aeruginosa*. *J. Exp. Med.* 196, 109-118.

Jeukens, J., Boyle, B., Kukavica-Ibrulj, I., Ouellet, M. M., Aaron, S. D., et al., (2014). Comparative genomics of isolates of a *Pseudomonas aeruginosa* epidemic strain associated with chronic lung infections of cystic fibrosis patients. *PLoS One*. 9, e87611. DOI: 10.1371/journal.pone.0087611

Jeukens, J., Kukavica-Ibrulj, I., Emond-Rheault, J. G., Freschi, L. and Levesque, R. C. (2017). Comparative genomics of a drug-resistant *Pseudomonas aeruginosa* panel and the challenges of antimicrobial resistance prediction from genomes. *FEMS Microbiol. Lett*. 364, fnx161. DOI: 10.1093/femsle/fnx161

Jeukens, J., Freschi, L., Kukavica-Ibrulj, I., Emond-Rheault, J. G., Tucker, N. P. and Levesque, R. C. (2019). Genomics of antibiotic-resistance prediction in *Pseudomonas aeruginosa*. *Ann N Y Acad Sci.* 1435, 5-17. doi: 10.1111/nyas.13358. Epub 2017 Jun 2. Review. PMID: 28574575 Free Article

Kidd, J. M., Kuti, J. L. and Nicolau, D. P. (2018), Novel pharmacotherapy for the treatment of hospital-acquired and ventilator-associated pneumonia caused by resistant gram-negative bacteria. *Expert Opin. Pharmacother*. 19, 397-408. DOI: 10.1080/14656566.2018.1438408

Martis, N., Leroy, S. and Blanc, V. (2014). Colistin in multi-drug resistant *Pseudomonas aeruginosa* blood-stream infections: a narrative review for the clinician. *J Infect.* 69, 1-12. doi: 10.1016/j.jinf.2014.03.001. Epub 2014 Mar 11

Mulani, M. S., Kamble, E. E., Kumkar, S. N., Tawre, M. S. and Pardesi, K. R. (2019). Emerging strategies to combat ESKAPE pathogens in the era of antimicrobial resistance: A Review. *Front. Microbiol.* 10, 539. DOI: 10.3389/fmicb.2019.00539

Potron, A., Poirel, L. and Nordmann, P. (2015). Emerging broad-spectrum resistance in *Pseudomonas aeruginosa* and *Acinetobacter baumannii*: Mechanisms and epidemiology. *Int J Antimicrob Agents.* 45, 568-585. doi: 10.1016/j.ijantimicag.2015.03.001. Epub 2015 Mar 24. Review. PMID: 25857949

Potron, A., Bour, M., Triponney, P., Muller, J., Koebel, C, A., et al., (2019). Sequential emergence of colistin and rifampicin resistance in an OXA-72- producing outbreak strain of *Acinetobacter baumannii*. *Int J Antimicrob Agents.* 53, 669-673. doi: 10.1016/j.ijantimicag.2019.01.012. Epub 2019 Jan 24. PMID: 30685310

Puzari, M. and Chetia, P. (2017). RND efflux pump mediated antibiotic resistance in Gram-negative bacteria *Escherichia coli* and *Pseudomonas aeruginosa*: a major issue worldwide. *World J. Microbiol. Biotechnol*. 33, 24. DOI: 10.1007/s11274-016-2190-5

Queenan, A. M. and Bush, K. (2007). Carbapenemases: the versatile beta-lactamases. *Clin. Microbiol. Rev*. 20, 440-458. DOI: 10.1128/CMR.00001-07

Subedi, D., Vijay, A. K. and Willcox, M. (2018). Overview of mechanisms of antibiotic resistance in *Pseudomonas aeruginosa*: an ocular perspective. *Clin. Exp. Optom*. 101, 162-171. DOI: 10.1111/cxo.12621

Nordmann, P., Ronco, E., Naas, T., Duport, C., Michel-Briand, Y. and Labia, R. (1993). Characterization of a novel extended-spectrum beta-lactamase from *Pseudomonas aeruginosa*. *Antimicrob. Agents Chemother*. 37, 962-969. DOI: 10.1128/AAC.37.5.962

Lupo, A., Haenni, M. and Madec, J. Y. (2018). Antimicrobial resistance in *Acinetobacter* spp. and *Pseudomonas* spp. *Microbiol. Spectr*. 6. DOI: 10.1128/microbiolspec

Nehme, D., Li, X. Z., Elliot, R. and Poole, K. (2004). Assembly of the MexAB-OprM multidrug efflux system of Ps*eudomonas aeruginosa*: identification and characterization of mutations in mexA compromising MexA multimerization and interaction with MexB

Nehme, D. and Poole, K. (2005). Interaction of the MexA and MexB components of the MexAB-OprM multidrug efflux system of *Pseudomonas aeruginosa*: identification of MexA extragenic suppressors of a T578I mutation in MexB. *Antimicrob. Agents Chemother*. 49, 4375-4378. DOI: 10.1128/AAC.49.10.4375-4378.2005

Pachori, P., Gothalwal, R. and Gandhi, P. (2019). Emergence of antibiotic resistance *Pseudomonas aeruginosa* in intensive care unit; a critical review. *Genes Dis*. 6, 109-119. DOI: 10.1016/j.gendis.2019.04.001

Romano, K. P., Warrier, T., Poulsen, B. E., Nguyen, P. H., Loftis, A. R., et al,, (2019). Mutations in pmrB confer cross-resistance between the LptD inhibitor POL7080 and colistin in *Pseudomonas* [Epub ahead of print] PMID: 31235628 Free Article

Szabó, D., Szentandrássy, J., Juhász, Z. S., Katona, K., Nagy, K. and Rókusz, L. (2008). Imported PER-1 producing *Pseudomonas aeruginosa*, PER-1 producing *Acinetobacter baumannii* and VIM-2-producing *Pseudomonas aeruginosa* strains in Hungary. *Ann. Clin. Microbiol. Antimicrob*. 7, 12. DOI: 10.1186/1476-0711-7-12

Vahaboglu, H., Coskunkan, F., Tansel, O., Ozturk, R., Sahin, N., Koksal, I., et al., (2001). Clinical importance of extended-spectrum beta-lactamase (PER-1-type)-producing *Acinetobacter* spp. and *Pseudomonas aeruginosa* strains. *J. Med. Microbiol*. 50, 642-645. DOI: 10.1099/0022-1317-50-7-642

Vila, J. and Martínez, J. L. (2008). Clinical impact of the over-expression of efflux pump in nonfermentative Gram-negative bacilli, development of efflux pump inhibitors. *Curr. Drug Targets* 9, 797-807.

Wozniak, T. M., Barnsbee, L., Lee, X. J. and Pacella, R. E. (2019). Using the best available data to estimate the cost of antimicrobial resistance: a systematic review. *Antimicrob. Resist. Infect. Control*. 8, 26. DOI: 10.1186/s13756-019-0472-z. eCollection 2019

Zhanel G. G., Lawrence CK, Adam H, Schweizer F, Zelenitsky S, Zhanel M, et al., (2018). Imipenem-Relebactam and Meropenem-Vaborbactam: Two Novel Carbapenem-β-Lactamase Inhibitor Combinations. *Drugs* 78, 65-98. doi: 10.1007/s40265-017-0851-9. Review. Erratum in: Drugs. 2018 May 10; PMID: 29230684

Supplementary Text Material 6

Antibiotic Resistance Mechanisms in *Acinetobacter baumannii*

The entry of '**Antibiotic resistance mechanisms in *Acinetobacter baumannii***' invoked 6,667 items (articles, including 3,740 reviews; 665 of which appeared in 2019) today, 08.28.2019.

As reviewed by Poirel et al (2011), in the genome of *A. baumannii*, there are two β-lactamase genes located. AmpC β-lactamases (Liu, and Liu, (2015); were found in China; coexistence of blaOXA-23 with blaNDM-1 and armA in clinical isolates of from India, (Karthikeyan, et al., (2010).

Imipenem resistance is due to altered penicillin-binding proteins, Gehrlein et al., 1991); of blaOXA-23-carrying imipenem-resistant recently was found in Taiwan, (Lin, M et al. 2011.) In *A. baumannii* there are weak permeability and strong efflux systems (Poole K. 2004; Vila et al., 2007; Peleg et al., 2008; Coyne et al., 2010; Coyne et al., 2011); Poirel et al., 2011; and later by Olajtan et al, 2014; Oliveira and Reygaert WC. (2019) altogether responsible for an intrinsic reduced susceptibility to antibiotics.

Furthermore, many acquired mechanisms of resistance have been identified in *A. baumannii*. (Kempf and Rolain, 2012; Olajtan et al, 2014; C.R. Lee et al., 2017; J. Y. Lee et al., 2017).

The MDR is the cumulative phenotype (reflecting the genotype); that is of expressed genes encoding for polypeptides (like *gyr*A, Vila et al. 1995, causing quinolone-resistance); or acting as decomposing enzymes, like carbapenemases; (Nowak and Paluchowska, 2016; Rodriguez et al., 2018); and/or porins; (Vila et al., 2007); and/or reduce permeability, (Oliveira and Reygaert, 2019; Li X. et al., 2019) and /or constitutive expression of active multi-substrate efflux systems (Peleg et al., 2008; Wieczorek, 2008; Laudy, (2018), Xu et al., 2019) expressed.
The antimicrobial resistance mechanisms in *A. baumannii* have been reviewed (Poirel et al., 2011; Kempf and Rolain, 2012; Peleg et al., 2012; Seiffert et al., 2013 (Blagg, 1967; Biswas et al., 2012; Dafopoulou et al., 2015A); Le Minh et al., 2015); or in combination with other antibiotics, (Nordqvist et al., 2016; Perez et al., 2018).

β-lactamases

Inactivation of β-lactams by β-lactamases is a major antibiotic resistance mechanism in *A. baumannii*. Based on sequence homology, β-lactamases are grouped into molecular classes, A, B, C, and D (Traglia et al., 2014). All four classes of β-lactamases were identified in *A. baumannii*. Recent studies have shown that it has natural competence to incorporate exogenous DNA, its genome has DNA of foreign origin at high frequencies and serum albumin enhances the natural competence of *A. baumannii* (Al-Agamy et al., 2014).

Since 2006, 17 Class A β-lactamases have been discovered and have been described (or re-described),

They are GES-1 and GES-5 (Chihi et al., 2016); GES-11 (Martinez et al., 2016); KPC-2 and (Aly et al., 2016); PER-1. The last, (like other pathogen bacteria such as Clostridium perfringens) is also an essential virulence factor needed to adhere A. baumannii cells to the target cell membrane (Voulgari et al., 2016).

Since 2006, 18 Class B β-lactamases have been described or re-described, including NDM-1 (Kumar, 2016) and NDM-3 (Bou and Martinez-Beltran, 2000), discovered in 2016. There is only 1 new Class A β-Lactamase, called AmpC, (Liu and Liu, 2015; Gonzalez-Villoria et al., 2016).

Forty-nine Class D (OXA) β-Lactamases have also been described from *A. baumannii;* 4 of them - (OXA-239, OXA-72, OXA-51, and OXA-253) - were discovered or re-described in the last year, (Dortet et al., 2016; Kuo et al., 2016; de Sa Cavalcanti et al., 2016; Fang et al., 2016; Vijayakumar et al., 2016; Biglari et al., 2017).

Carbapenemases

One report from India showed that blaOXA-51 and blaOXA-23 were present in all 103 carbapenem-resistant *A. baumannii* isolates. Almost 80% of the isolates had ISAba1 upstream of the blaOXA-23 ases. The latest review on *A. baumannii* carbapenemases was published last year, (Rodríguez et al., 2018).

Aminoglycoside-modifying enzymes

They are the major resistance mechanism in A. baumannii to neutralize aminoglycosides. They are all encoded by genes localized in mobile genetic elements in different isolates of the *A. baumannii* species (Bouvet and Grimont, 1986).

Aminoglycoside acetyltransferases are represented by 5 enzymes: AAC3 (Doi et al., 2004), aaC1, aac2, AAC (6'), aacA4 (Gallego and Towner, 2001; Cho et al., 2009; Zhu et al., 2009; Lin et al., 2011b; Lin et al., 2013; Bakour et al., 2014). The respective genes were identified by Wen et al., 2014

Aminoglycoside adenyltransferases are represented by 4 enzymes: ANT (2″), (aadB), (Doi et al., 2004), ANT (3″) and (aadA1), (Zhu et al., 2009; Bakour et al., 2014).

Aminoglycoside phosphotransferases are represented by 3 enzymes: PH (3′), (aphA1) (Peleg et al., 2007) and APH (3″) (Liu and Liu, 2015).

Efflux pumps

In *A. baumannii*, efflux pumps are associated with resistance against many different classes of antibiotics, such as tigecycline (Hu et al., 2007) and imipenem (Deng et al., 2014). Loss of antibiotic resistance invoked by efflux pump inhibitors such as 1-(1-naphthylmethyl)-piperazine and carbonyl cyanide 3-chlorophenyl-hydrazone is supporting evidence of the importance of efflux pumps (Magnet et al., 2001).

As for efflux pumps, the resistance-nodulation-division efflux pump superfamily is represented by 3 members in A. baumannii. The AdeABC is responsible for aminoglycoside resistance [183] (Ruzin et al., 2007), and for the reduced susceptibility to tigecycline (Higgins et al., 2013) and to non-fluoroquinolone antibiotics Lin et al., 2014). The responsible gene is repressed in the wild type by the BaeSR two-component system (Lin et al., 2015; Sun et al., 2016) but over-expressed in the respective ("inducer") mutants [(Damier-Piolle et al., 2008).

AdeFGH (He et al., 2015) and AdeIJK (Coyne et al., 2010) are synergistically associated with tigecycline resistance (Coyne et al., 2010). AdeFGH and AdeIJK expression is regulated by TetR-type transcriptional regulator AdeN (Ribera et al., 2003; Rosenfeld et al., 2012;).

The major facilitator efflux pump superfamily is represented by TetA [(Vilacoba et al., 2013), TetB (Coyne et al., 2011) and CmlA (Roca et al., 2009), CraA (Rajamohan et al., 2010) (responsible for tetracycline and chloramphenicol resistance, respectively), AmvA (mediating resistance towards different classes of molecules of antibacterial activity, such as disinfectants, detergents, and dyes, furthermore erythromycin, acriflavine, benzalkonium chloride, and methylviologen) (Sharma et al., 2016), and finally, AbaF (responsible for fosfomycin resistance) (Su et al., 2005).

Multi-drug and toxic compound extrusion family represented by AbeM is responsible for resistance to imipenem and fluoroquinolones (Srinivasan et al., 2015). Deletion mutants of the small multidrug resistance family AbeS show increased sensitivities to different antibiotics (Nowak-Zaleska et al., 2016).

The EmrAB-TolC efflux pump is also present in A. baumannii and responsible for resistance to netilmicin, tobramycin and imipenem (Li et al., 2016). A1S-1535 confers resistance to gentamicin, kanamycin, chloroxylenol, oxytetracycline, 1, 10-phenanthroline, and chloramphenicol (Bou et al., 2001). A1S-2795 is responsible for resistance to the sulphonamide sulfathiazole, and ABAYE-0913 is associated with resistance to chloramphenicol and fusidic acid (Bou et al., 2001).

Altered permeability resulting in antibiotic resistance in *A. baumannii*

Porins which form channels that allow transport of molecules across the outer membrane of Gram-negative bacteria, resulted in carbapenem resistance in *A. baumannii* (Diancourt et al., 2010). Carbapenem resistance could be a phenotypic consequence of reduced expressions of some porins, such as Omp22-33 (Mussi et al., 2007), or CarO (Catel-Ferreira et al., 2011; Fonseca et al., 2013). Imipenem resistance could be the phenotypic consequence of the loss of Omp29, producing OXA-51-like, or OXA-23-like, carbapenemases (Smani et al., 2014). Aztreonam, chloramphenicol, and nalidixic acid resistance is related with OmpA (Wu et al., 2016). OmpA and CarO have recently been reported as being associated with antibiotic resistance through physical interactions with OXA-23 carbapenemase (Gehrlein et al., 1991). Alteration of target sites is the resistance mechanism materialized by modifications in antibiotic target sites in A. baumannii. Alteration of target sites seems to be a successful mechanism against almost all antibiotics tested so far. Imipenem resistance could be the phenotypic consequences of overexpression of altered penicillin-binding proteins (PBPs), which have a low affinity for imipenem (Yu et al., 2007). Aminoglycoside resistance could be the phenotypic consequences of the 16S rRNA methylase (ArmA) activity, found in several pathogen isolates. It always coexists with OXA-type carbapenemases such as OXA-23 (Vila et al., 1995; Gallego and Towner, 2001; Karthikeyan et al., 2010; Brigante et al., 2012; Hong et al., 2013; Tada et al., 2014; Hasani et al., 2016). Quinolone resistance could be the phenotypic consequence of modifications in GyrA – coding structure gene (GyrA is one subunit of DNA gyrase), and that of ParC (one subunit of topoisomerase IV), in epidemiologically unrelated A. baumannii isolates (Ribera et al., 2003). Tetracycline resistance, determined by TetM, is thought to act through ribosomal protection (Mák et al., 2009). Trimethoprim resistance has been found in nosocomial MDR *A. baumannii* isolates and is supposed be a phenotypic consequence of the action of dihydrofolate reductases (DHFR and FolA), (de Breij et al., 2010; Taitt et al., 2014; Kuo et al., 2016). Other Resistance Mechanisms are also discussed by Lee et al. (2017).

The BaeSR Two-Component Regulatory System The BaeSR Two-Component Regulatory

System (discovered in E. coli, Baranova and Nikkaido, 2002) also found in *A. baumannii*,

REFERENCES

Al-Agamy, M. H., Jeannot, K., El-Mahdy, T. S., Shibl, A. M., Kattan, W., Plésiat, P. and Courvalin, P. (2014). First detection of GES-5 carbapenemase producing *Acinetobacter baumannii* Isolate. *Microb. Drug Resist*. 23, 556-562. DOI: 10.1089/mdr.2016.0152.
Aly, M. M., Abu Alsoud, N. M., Elrobh, M. S., Al Johani, S. M. and Balkhy, H. H. (2016). High prevalence of the PER-1 gene among carbapenem-resistant Acinetobacter baumannii in Riyadh, Saudi Arabia. *Eur. J. Clin. Microbiol. Infect Dis.* 35, 1759-1766. DOI: 10.1007/s10096-016-2723-8.
Bakour, S., Alsharapy, S. A., Touati, A. and Rolain J. M. (2014). Characterization *of Acinetobacter baumannii* clinical isolates carrying blaOXA-23carbapenemase and 16S rRNA methylase armA genes in Yemen. *Microb. Drug Resist*. 20, 604-609. DOI: 10.1089/mdr.2014.0018.
Baranova, N. and Nikaido, H. (2002). The BaeSR Two-Component Regulatory System Activates Transcription of the yegMNOB (mdtABCD) Transporter Gene Cluster in *Escherichia coli* and Increases Its Resistance to Novobiocin and Deoxycholate *J Bacteriol*. 184, 4168-4176.doi: 10.1128/JB.184.15.4168-4176.2002 PMCID: PMC135214 PMID: 12107134

Biglari, S., Hanafiah, A., Mohd Puzi, S., Ramli, R., Rahman, M. and Lopes, B. S. (2017). Antimicrobial resistance mechanisms and genetic diversity of multidrug-resistant *Acinetobacter baumannii* isolated from a teaching hospital in Malaysia. *Microb. Drug Resist*. 23, 545-555. DOI: 10.1089/mdr.2016.0130.
Bou, G., Cerveró, G., Angeles Domínguez, M., Quereda, C. and Martínez-Beltrán, J. (2001). Characterization of a nosocomial outbreak caused by a multiresistant *Acinetobacter baumannii* strain with a carbapenem-hydrolyzing enzyme: High-level carbapenem resistance in *A. baumannii* is not due solely to the presence of β-lactamases. *J. Clin. Microbiol*. 38, 3299-3305
Bou, G. and Martinez-Beltran, J. (2000). Cloning, nucleotide sequencing, and analysis of the gene encoding an AmpC β-lactamases in *Acinetobacter baumannii*. *Antimicrob. Agents Chemother*. 44, 428-432. DOI: 10.1128/AAC.44.2.428-432.2000

Chihi, H., Bonnin, R. A., Bourouis, A., Mahrouki, S., Besbes, S., Moussa, M. B., Belhadj, O. and Naas, T. (2016). GES-11-producing *Acinetobacter baumannii* clinical isolates from Tunisian hospitals: long-term dissemination of GES-type carbapenemases in North Africa. *J. Glob. Antimicrob. Resist.* 5, 47-50. DOI: 10.1016/j.jgar.2016.03.005.

Cho, Y. J., Moon, D. C., Jin, J. S., Choi, C. H., Lee, Y. C. and Lee, J. C. (2009). Genetic basis of resistance to aminoglycosides in *Acinetobacter* spp. and spread of armA in *Acinetobacter baumannii* sequence group 1 in Korean hospitals. *Diagn. Microbiol. Infect. Dis*. 64, 185-190. DOI: 10.1016/j.diagmicrobio.2009.02.010.
Coyne, S., Rosenfeld, N., Lambert, T., Courvalin, P. and Perichon, B. (2010). Overexpression of resistance-nodulation-cell division pump AdeFGH confers multidrug resistance in *Acinetobacter baumannii*. *Antimicrob. Agents Chemother*. 54, 4389-4393. DOI: 10.1128/AAC.00155-10.
Coyne, S., Courvalin, P. and Perichon, B. (2011). Efflux-mediated antibiotic resistance in *Acinetobacter* spp. *Antimicrob. Agents Chemother*. 55, 947-953. DOI: 10.1128/AAC.01388-10

Damier-Piolle, L., Magnet, S., Bremont, S., Lambert, T. and Courvalin, P. (2008). AdeIJK, a resistance-nodulation-cell division pump effluxing multiple antibiotics in *Acinetobacter*

baumannii. *Antimicrob. Agents Chemother*. 52, 557-562. DOI: 10.1128/AAC.00732-07.
de Sa Cavalcanti, F. L., Mendes-Marques, C. L., Vasconcelos, C. R., de Lima Campos, T.., Rezende, A. M., Xavier, D. E., Leal, N. C., de-Melo-Neto, O. P., de Morais, M. M. and Leal-Balbino, T. C. (2016). High frequency of OXA-253-producing *Acinetobacter baumannii* in different hospitals in Recife, Brazil: a new threat? *Antimicrob. Agents Chemother*. 61:e01309-16. DOI: 10.1128/AAC.01309-16.
Deng, M., Zhu, M. H., Li, J. J., Bi, S., Sheng, Z. K., Hu, F. S., Zhang, J. J., Chen, W., Xue, X. W., Sheng, J. F. and Li, L. J. (2014). Molecular epidemiology and mechanisms of tigecycline resistance in clinical isolates of *Acinetobacter baumannii* from a Chinese university hospital. *Antimicrob. Agents Chemother*. 58, 297-303. DOI: 10.1128/AAC.01727-13
Doi, Y., Wachino, J., Yamane, K., Shibata, N., Yagi, T., Shibayama, K., Kato, H. and Arakawa, Y. (2004). Spread of novel aminoglycoside resistance gene aac(6′)-Iad among *Acinetobact*er clinical isolates in Japan. *Antimicrob. Agents Chemother*. 48, 2075-2080. DOI: 10.1128/AAC.48.6.2075-2080.2004.
Dortet, L., Bonnin, R. A., Bernabeu, S., Escaut, L., Vittecoq, D., Girlich, D., Imanci, D., Fortineau, N. and Naas, T. (2016). First occurrence of OXA-72-producing *Acinetobacter baumannii* in Serbia. *Antimicrob. Agents Chemother*. 60, 5724-5730. DOI: 10.1128/AAC.01016-16.
Fang, F., Wang, S., Dang, Y. X., Wang, X. and Yu, G. Q. (2016). Molecular characterization of carbapenemase genes in *Acinetobacter baumannii* in China. *Genet. Mol. Res*. 15:gmr.15017432. DOI: 10.4238/gmr.15017432.
Gallego, L. and Towner, K. J. (2001). Carriage of class 1 integrons and antibiotic resistance in clinical isolates of *Acinetobacter baumannii* from northern Spain. *J. Med. Microbiol*. 50, 71-77. DOI: 10.1099/0022-1317-50-1-71.
Gehrlein, M., Leying, H., Cullmann, W., Wendt, S. and Opferkuch, W. (1991). Imipenem resistance in *Acinetobacter baumannii* is due to altered penicillin-binding proteins. *Chemotherapy*. 37, 405-412. DOI: 10.1159/000238887

Gonzalez-Villoria, A. M., Tamayo-Legorreta, E., Garza-Ramos, U., Barrios, H., Sanchez-Perez, A., Rodriguez-Medina, N., Uribe-Aviña, N., Cevallos, M. A. and Silva-Sanchez, J. (2016). A multicenter study in Mexico finds *Acinetobacter baumannii* clinical isolates belonging to clonal complexes 636B (113B) and 92B harboring OXA-72, OXA-239, and OXA-469. *Antimicrob. Agents Chemother*. 60, 2587-2588. DOI: 10.1128/AAC.02042-15.
Higgins, P. G., Perez-Llarena, F. J., Zander, E., Fernandez, A., Bou, G. and Seifert, H. (2013). OXA-235, a novel class D β-lactamase involved in resistance to carbapenems in *Acinetobacter baumannii*. *Antimicrob. Agents Chemother*. 57, 2121-2126. DOI:10.1128/AAC.02413-12.
He, X., Lu, F., Yuan, F., Jiang, D., Zhao, P., Zhu, J., Cheng, H., Cao, J. and Lu, G. (2015). Biofilm formation caused by clinical *Acinetobacter baumannii* isolates is associated with overexpression of the AdeFGH efflux pump. *Antimicrob. Agents Chemother*. 59, 4817-4825. DOI: 10.1128/AAC.00877-15.
Hu, W. S., Yao, S. M., Fung, C. P., Hsieh, Y. P., Liu, C. P. and Lin J. F. (2007). An OXA-66/OXA-51-like carbapenemase and possibly an efflux pump are associated with resistance to imipenem in *Acinetobacter baumannii*. *Antimicrob. Agents Chemother*. 51, 3844-3852. DOI: 10.1128/AAC.01512-06.
Karthikeyan, K., Thirunarayan, M. A. and Krishnan, P. (2010). Coexistence of blaOXA-23 with blaNDM-1 and armA in clinical isolates of *Acinetobacter baumannii* from India. *J. Antimicrob. Chemother*. 65, 2253-2254. DOI: 10.1093/jac/dkq273

Kempf, M. and Rolain, J. M. (2012). Emergence of resistance to carbapenems in *Acinetobacter baumannii* in Europe: clinical impact and therapeutic options. *Int. J. Antimicrob. Agents*. 39, 105-114. DOI: 10.1016/j.ijantimicag.2011.10.004

Kumar, M. (2016). Identification of a novel NDM variant, *blaNDM−3*, from a multidrug-resistant *Acinetobacter baumannii*. *Infect. Control. Hosp. Epidemiol*. 37, 747-748. DOI: 10.1017/ice.2016.66.

Kuo, H. Y., Hsu, P. J., Chen, J. Y., Liao, P. C., Lu, C. W., Chen, C. H. and Liou, M. L. (2016).). Clonal spread of blaOXA-72-carrying *Acinetobacter baumannii* sequence type 512 in Taiwan. *Int. J. Antimicrob. Agents*. 48, 111-113. DOI: 10.1016/j.ijantimicag.2016.04.020.

Lee, C. R., Lee, J. H., Park, M., Park, K. S., Bae, I. K., et al., (2017). Biology of *Acinetobacter baumannii*: Pathogenesis, antibiotic resistance mechanisms, and prospective treatment options. *Front. Cell. Infect. Microbiol*. 7, 55. DOI: 10.3389/fcimb.2017.00055

Lee, J. -Y, Chung, E. S. and Ko, K. S. (2017). Transition of colistin dependence into colistin resistance in *Acinetobacter baumannii. Sci. Rep.* 7, 14216. DOI: 0.1038/s41598-017-14609-0

Li, L., Hassan, K. A., Brown, M. H. and Paulsen, I. T. (2016). Rapid multiplexed phenotypic screening identifies drug resistance functions for three novel efflux pumps in *Acinetobacter baumannii*. *J. Antimicrob. Chemother*. 71, 1223-1232. DOI: 10.1093/jac/dkv460

Lin, M. F., Kuo, H. Y., Yeh, H. W., Yang, C. M., Sung, C. H., Tu, C. C., Huang, M. L. and Liou, M. L. (2011b). Emergence and dissemination of blaOXA-23-carrying imipenem-resistant *Acinetobacter* sp. in a regional hospital in Taiwan. *J. Microbiol. Immunol. Infect*. 44, 39-44. DOI:10.1016/j.jmii.2011.01.008.

Lin, M. F., Liou, M. L., Tu, C. C., Yeh, H. W. and Lan, C. Y. (2013). Molecular epidemiology of integron-associated antimicrobial gene cassettes in the clinical isolates of *Acinetobacter baumannii* from northern Taiwan. *Ann. Lab. Med*. 33, 242-247. DOI: 10.3343/alm.2013.33.4.242

Liu, Y. and Liu, X. (2015). AmpC detection of AmpC β-lactamases in *Acinetobacter baumannii* in the Xuzhou region and analysis of drug resistance. *Exp. Ther. Med*. 10, 933-936. DOI: 10.3892/etm.2015.2612.

Lin, M. F., Lin, Y. Y., Yeh, H. W. and Lan, C. Y. (2014). Role of the BaeSR two-component system in the regulation of *Acinetobacter baumannii* adeAB genes and its correlation with tigecycline susceptibility. *BMC Microbiol*. 14, 119. DOI: 10.1186/1471-2180-14-119.

Lin, M. F., Lin, Y. Y. and Lan, C. Y. (2015). The role of the two-component system BaeSR in disposing chemicals through regulating transporter systems in *Acinetobacter baumannii*. *PLoS ONE*. 10:e0132843. DOI: 10.1371/journal.pone.0132843.

Magnet, S., Courvalin, P. and Lambert, T. (2001). Resistance-nodulation-cell division-type efflux pump involved in aminoglycoside resistance in *Acinetobacter baumannii* strain BM4454. *Antimicrob. Agents Chemother*. 45, 3375-3380. DOI: 10.1128/AAC.45.12.3375-3380.2001.

Martinez, T., Martinez, I., Vazquez, G. J., Aquino, E. E. and Robledo, I. E. (2016). Genetic environment of the KPC gene in *Acinetobacter baumannii* ST2 clone from Puerto Rico and genomic insights into its drug resistance. *J. Med. Microbiol*. 65, 784-792. DOI: 10.1099/jmm.0.000289.

Nemec, A., Dolzani, L., Brisse, S., van den Broek, P. and Dijkshoorn L. (2004). Diversity of aminoglycoside-resistance genes and their association with class 1 integrons among strains of pan-European *Acinetobacter baumannii* clones. *J. Med. Microbiol*. 53, 1233-1240. DOI: 10.1099/jmm.0.45716-0.

Nowak-Zaleska, A., Wieczor, M., Czub, J., Nierzwicki, L., Kotlowski, R., Mikucka, A., Gospodarek, E. (2016). Correlation between the number of Pro-Ala repeats in the EmrA homologue of *Acinetobacter baumannii* and resistance to netilmicin, tobramycin, imipenem and ceftazidime. *J. Glob. Antimicrob. Resist.* 7, 145-149. DOI: 10.1016/j.jgar.2016.09.004.

Olaitan, A. O., Morand, S. and Rolain, J. M. (2014). Mechanisms of polymyxin resistance: acquired and intrinsic resistance in bacteria. *Front. Microbiol*. 5, 643. DOI: 10.3389/fmicb.2014.00643

Oliveira J, Reygaert WC. (2019). Gram negative bacteria StatPearls [Internet]. Treasure Island (FL): StatPearls Publishing; 2019 Jan-.2019 Mar 9. PMID: 30855801 Free Books & Documents

Peleg, A. Y., Adams, J. and Paterson, D. L. (2007). Tigecycline efflux as a mechanism for nonsusceptibility in *Acinetobacter baumannii*. *Antimicrob. Agents Chemother*. 51, 2065-2069. DOI: 10.1128/AAC.01198-06.

Peleg, A. Y., Seifert, H. and Paterson, D. L. (2008). *Acinetobacter baumannii*: emergence of a successful pathogen. *Clin. Microbiol. Rev*. 21, 538-582. DOI: 10.1128/CMR.00058-07

Poirel, L., Bonnin, R. A. and Nordmann, P. (2011). Genetic basis of antibiotic resistance in pathogenic Acinetobacter species. *IUBMB Life.* 63, 1061-1067. DOI: 10.1002/iub.532

Poole K. (2004). Efflux-mediated multiresistance in Gram-negative bacteria. *Clin Microbiol Infect.* 10, 12-26. Review. PMID: 14706082 Free Article

Rajamohan, G., Srinivasan, V. B. and Gebreyes, W. A. (2010). Molecular and functional characterization of a novel efflux pump, AmvA, mediating antimicrobial and disinfectant resistance in *Acinetobacter baumannii*. *J. Antimicrob. Chemother*. 65, 1919-1925. DOI: 10.1093/jac/dkq195.

Ribera, A., Roca, I., Ruiz, J., Gibert, I. and Vila, J. (2003). Partial characterization of a transposon containing the tet(A) determinant in a clinical isolate of Acinetobacter baumannii. J. Antimicrob. Chemother. 52, 477-480. DOI: 10.1093/jac/dkg344

Roca, I., Marti, S., Espinal, P., Martinez, P., Gibert, I. and Vila, J. (2009). CraA, a major facilitator superfamily efflux pump associated with chloramphenicol resistance in *Acinetobacter baumannii*. *Antimicrob. Agents Chemother*. 53, 4013-4014. DOI: 10.1128/AAC.00584-09.

Rosenfeld, N., Bouchier, C., Courvalin, P. and Perichon B. (2012). Expression of the resistance-nodulation-cell division pump AdeIJK in *Acinetobacter baumannii* is regulated by AdeN, a TetR-type regulator. *Antimicrob. Agents Chemother*. 56, 2504. DOI: 10.1128/AAC.06422-1.

Ruzin, A., Keeney, D. and Bradford, P. A. (2007). AdeABC multidrug efflux pump is associated with decreased susceptibility to tigecycline in Acinetobacter calcoaceticus-Acinetobacter baumannii complex. *J. Antimicrob. Chemother*. 59, 1001-1004. DOI: 10.1093/jac/dkm058.

Sharma, A., Sharma, R., Bhattacharyya, T., Bhando, T. and Pathania, R. (2016). Fosfomycin resistance in Acinetobacter baumannii is mediated by efflux through a major facilitator superfamily (MFS) transporter-AbaF. *J. Antimicrob. Chemother*. 72, 68-74. DOI: 10.1093/jac/dkw382.

Srinivasan, V. B., Venkataramaiah, M., Mondal, A. and Rajamohan, G. (2015). Functional characterization of AbeD, an RND-type membrane transporter in antimicrobial resistance in *Acinetobacter baumannii*. *PLoS ONE* 10:e0141314. DOI: 10.1371/journal.pone.0141314.

Su, X. Z., Chen, J., Mizushima, T., Kuroda, T. and Tsuchiya, T. (2005). AbeM, an H+-coupled Acinetobacter baumannii multidrug efflux pump belonging to the MATE family of transporters. *Antimicrob. Agents Chemother*. 49, 4362-4364. DOI: 10.1128/AAC.49.10.4362-4364.2005.

Sun, J. R., Jeng, W. Y., Perng, C. L., Yang, Y. S., Soo, P. C., Chiang. Y. S. and Chiueh, T. S. (2016). Single amino acid substitution Gly186Val in AdeS restores tigecycline susceptibility of *Acinetobacter baumannii*. J. *Antimicrob. Chemother.* 71, 1488-1492. DOI: 10.1093/jac/dkw002..

Tada, T., Miyoshi-Akiyama, T., Shimada, K., Shimojima, M. and Kirikae, T. (2014). Dissemination of 16S rRNA methylase ArmA-producing *Acinetobacter baumannii* and emergence of OXA-72 carbapenemase coproducers in Japan. Antimicrob. Agents Chemother. 58, 2916-2920. DOI: 10.1128/AAC.01212-13

Taitt, C. R., Leski, T. A., Stockelman, M. G., Craft, D. W., Zurawski, D.   et al., V., Kirkup, B. C. and Vora, G. J. (2014). Antimicrobial resistance determinants in *Acinetobacter baumannii* isolates taken from military treatment facilities. Antimicrob. Agents Chemother. 58, 767-781. DOI: 10.1128/AAC.01897-13

Traglia, G. M., Chua, K., Centrón, D., Tolmasky, M. E. and Ramírez, M. S. (2014). Whole-genome sequence analysis of the naturally competent Acinetobacter baumannii clinical isolate A118. *Genome Biol. Evol.* 6, 2235-2239. DOI: 10.1093/gbe/evu176.

Vijayakumar, S., Gopi, R., Gunasekaran, P., Bharathy, M., Walia, K., Anandan, S. and Veeraraghavan, B. (2016). Molecular characterization of invasive carbapenem-resistant *Acinetobacter baumannii* from a tertiary care hospital in South India. *Infect. Dis. Ther.* 5, 379-387.

Vila, J., Martı, S. and Sánchez-Céspedes, J. (2007). Porins, efflux pumps and multidrug resistance in *Acinetobacter baumannii*. *J. Antimicrob. Chemother.* 59, 1210- 1215. DOI:10.1093/jac/dkl509

Vilacoba, E., Almuzara, M., Gulone, L., Traglia, G. M., Figueroa, S. A., Sly, G., Fernández, A., Centrón, D. and Ramírez, M. S. (2013). Emergence and spread of plasmid-borne tet(B)::ISCR2 in minocycline-resistant *Acinetobacter baumannii* isolates. *Antimicrob. Agents Chemother.* 57, 651-654. DOI: 10.1128/AAC.01751-12.

Voulgari, E., Politi, L., Pitiriga, V., Dendrinos, J., Poulou, A., Georgiadis, G. and Tsakris, A. (2016). First report of an NDM-1 metallo-β-lactamase-producing Acinetobacter baumannii clinical isolate in Greece. *Int. J. Antimicrob. Agents*. 48, 761-762. DOI: 10.1016/j.ijantimicag.2016.09.006.

Zhu, J., Wang, C., Wu, J., Jiang, R., Mi, Z. and Huang, Z. (2009).   A novel aminoglycoside-modifying enzyme gene aac(6′)-Ib in a pandrug-resistant *Acinetobacter baumannii* strain. *J. Hosp. Infect.* 73, 184-185. DOI: 10.1016/j.jhin.2009.05.012.

Biofilm Formation

Biofilm formation plays an important role not only in the immune evasion by *A. baumannii* (Dhabaan et al., 2015) but persistence as well. Imipenem treatment of the imipenem-resistant *A. baumannii* isolate induces expression of important genes responsible for the synthesis of type IV

pili (Rouli et al., 2015), the existence of which is needed for biofilm formation.

REFERENCES

Dhabaan, G. N., Abu-Bakr, S., Cerqueira, G. M., Al-Haroni, M., Pang, S. P. and Hassan, H. (2015). Imipenem treatment induces expression of important genes and phenotypes in a resistant *Acinetobacter baumannii* isolate. *Antimicrob. Agents Chemother*. 60, 1370-1376. DOI:10.1128/AAC.01696-15.

Rouli, L., Merhej, V., Fournier, P. -E. and Raoult, D. (2015). The bacterial pangenome as a new tool for analyzing pathogenic bacteria. *New. Microbes. New. Infect*. 7, 72-85. DOI: 10.1016/j.nmni.2015.06.005.

Virulence Factors of *A. baumannii*

The differences in mortality are probably not independent of the expression of specific virulence factors and determinants, (Dijkshoorn et al., 2007). The virulence factors of *A. baumannii* have been identified as targets of host innate pattern-recognition receptors, which leads to activation of downstream inflammasomes to develop inflammatory responses, and the recruitment of innate immune effectors against the pathogen. To inactivate the host immune attack, *A. baumannii* regulates its expression of different virulence factors. like porins, capsular polysaccharides, lipopolysaccharides, phospholipases, outer membrane vesicles, metal acquisition systems, and protein secretion systems, (Diancourt et al., 2010; Li FJ et al., 2018), which are not under the scope of this review.

REFRENCES

Dijkshoorn, L., Nemec, A. and Seifert, H. (2007). An increasing threat in hospitals: multidrug resistant *Acinetobacter baumannii*. *Nat. Rev. Microbiol.* 5, 939-951.

Diancourt, L., Passe, V., Nemec, A., Dijkshoorn, L. and Brisse, S. (2010). The population structure of *Acinetobacter baumannii*: expanding multiresistant clones from an ancestral susceptible genetic pool. *PLoS ONE*. 5: e10034. DOI: 10.1371/journal.pone.0010034

Li, F. J,, Starrs, L. and Burgio, G. (2018). Tug of war between *Acinetobacter baumannii* and host immune responses. *Pathog Dis.* 76(9). pii: ftz004. doi: 10.1093/femspd/ftz004.

Acinetobacter baumannii Caused Diseases and Pathomechanisms

A Grouped List of Recent Reviews on Diseases Caused by *A. baumannii*

The mechanisms of disease caused by *A. baumannii* strains have recently been reviewed in *Frontiers Microbiology* by Morris et al., 2019). The different *A. baumannii* strains could be scored according to their pathogenic potentials, (de Breij et al., 2012; C.R. Lee et al., 2017). The resistance panel and the capability of biofilm formation influence the pathogenic potentials of the different strains, de Breij et al., 2010; C.R. Lee et al., 2017). The first review on *Acinetobacter*-caused diseases appeared in 2007, (Dijkshoorn et al., 2007).

Diseases:

One group of several serious or fatal diseases are scored as pneumonia, (Eveillard et al., 2010; Serota et al., 2018; Bonell et al., 2019); including ventilator-associated pneumonia, (Cisneros-Herreros et al., 2005; Falagas and Rafailidis, 2007; Fragkou et al., 2019). The host innate immune response seems to determine morbidity and mortality in experimental pneumonia, (de Breij et al., 2012). Community-acquired *A. baumannii* pneumonia cases (Dexter et al., 2015) are even more fatal than nosocomial ones, (Bergogne–Bérézin and Towner, 1996; Serota et al., 2018). Somewhat rarer diseases are secondary meningitis (Ceylan et al., 2017). In a case report, intraventricular and intravenous tigecycline therapy was successfully used (Long et al. 2018), despite tigecycline efflux is a mechanism for nonsusceptibility by *A. baumannii*, (Peleg et al., 2007). Endocarditis (Chen et al., 2015) and bacteremia (Garnacho-Montero 2015; Alhashem et al., 2017; Casallas et al. 2019) are also on the "crime-list" of *A. baumannii.* related to *A. baumannii infections colistin treatments and of colistin-related nephrotoxicity are discussed by* Inci et al., 2018). Amongst infections, with high mortality rates, the recipient-born blood-stream infections, (Falagas and Rafailidis, 2007; Daskalaki et al., 2014; Andini et al., 2015), skin (Chim et al., 2007) soft tissue wound infections, (Pfalzgraff et al., 2018); and urinary tract infections, (Daskalaki et al., 2014; Di Venanzio et al., 2019) related to *A. baumannii* infections colistin treatments and of colistin-related nephrotoxicity are discussed by Inci et al., 2018).

References

Bergogne–Bérézin, E. and Towner, K. J. (1996). *Acinetobacter* spp. as nosocomial pathogens: microbiological, clinical, and epidemiological features. *Clin. Microbiol. Rev.* 9, 148-165

Alhashem, F., Tiren-Verbeet, N. L., Alp, E. and Doganay, M. (2017). Treatment of sepsis: What is the antibiotic choice in bacteremia due to carbapenem resistant Enterobacteriaceae? *World J. Clin. Cases* 5, 324-332. DOI: 10.12998/wjcc.v5.i8.324

Andini, R., Agrusta, F., Mattucci, I., Malgeri, U., Cavezza, G., et al., (2015). Recipient-born bloodstream infection due to extensively drug-resistant *Acinetobacter baumannii* after emergency heart transplant: report of a case and review of the literature. *Infection* 43, 609-13. DOI: 10.1007/s15010-015-0772-z

Bonell, A., Azarrafiy, R., Huong, V. T. L., Viet, T. L., Phu, V. D., et al., (2019). A systematic review and meta-analysis of ventilator-associated pneumonia in adults in Asia: An analysis of national income level on incidence and etiology. *Clin. Infect. Dis*. 68, 511-518. DOI: 10.1093/cid/ciy543 Bergogne–Bérézin, E. and Towner, K. J. (1996). *Acinetobacter* spp. as

nosocomial pathogens: microbiological, clinical, and epidemiological features. *Clin. Microbiol. Rev.* 9, 148-165

Bonell, A., Azarrafiy, R., Huong, V. T. L., Viet, T. L., Phu, V. D., et al., (2019). A systematic review and meta-analysis of ventilator-associated pneumonia in adults in Asia: An analysis of national income level on incidence and etiology. *Clin. Infect. Dis*. 68, 511-518. DOI: 10.1093/cid/ciy543

de Breij, A., Dijkshoorn, L., Lagendijk, E., van der Meer, J., Koster, A., Bloemberg, G., et al., (2010). Do biofilm formation and interactions with human cells explain the clinical success of *Acinetobacter baumannii*? *PLoS One* 5:e10732. DOI: 10.1371/journal.pone.0010732

de Breij, A., Eveillard, M., Dijkshoorn, L., van den Broek, P. J., Nibbering, P. H. and Joly-Guillou, M. L. (2012). Differences in *Acinetobacter baumannii* strains and host innate immune response determine morbidity and mortality in experimental pneumonia. *Plos One.* 7:e30673. DOI: 10.1371/journal.pone.0030673

Casallas, J. C. G., Robayo-Amortegui, H., Corredor-Rozo, Z., Carrasco-Márquez, A. M. and Escobar-Perez, J. (2019) Bacteremia by colistin-resistant *Acinetobacter baumannii* isolate: a case report. *J. Med. Case Rep*. 13, 141. DOI: 10.1186/s13256-019-2062-3

Ceylan, B., Arslan, F., Sipahi, O. R., Sunbul, M., Ormen, B., et al., (2017). Variables determining mortality in patients with *Acinetobacter baumannii* meningitis/ventriculitis treated with intrathecal colistin. *Clin. Neurol. Neurosurg*. 153, 43-49. DOI: 10.1016/j.clineuro.2016.12.006

Chen, Q., Cao, H., Lu, H., Qiu, Z. H. and He, J. J. (2015). Bioprosthetic tricuspid valve endocarditis caused by *Acinetobacter baumannii* complex, a case report and brief review of the literature. *J. Cardiothorac. Surg*. 10, 149. DOI: 10.1186/s13019-015-0377-8

Chim, H., Tan, B. H. and Song, C. (2007). Five-year review of infections in a burn intensive care unit: High incidence of *Acinetobacter baumannii* in a tropical climate. *Burns*. 33, 1008-1014. DOI: 10.1016/j.burns.2007.03.003

Daskalaki, E., Koukoulaki, M., Bakalis, A., Papastamopoulos, V., Belesiotou, E., et al., (2014). Blood stream infections in renal transplant recipients: a single-center study. *Transplant Proc*. 46, 3191-3193. DOI: 10.1016/j.transproceed.2014.10.033

Dexter, C., Murray, G. L., Paulsen, I. T. and Peleg, A. Y. (2015). Community-acquired *Acinetobacter baumannii*: clinical characteristics, epidemiology and pathogenesis. *Expert Rev. Anti. Infect. Ther*. 13, 567-753. DOI: 10.1586/14787210.2015.1025055

Dijkshoorn, L., Nemec, A. and Seifert, H. (2007). An increasing threat in hospitals: multidrug resistant *Acinetobacter baumannii*. *Nat. Rev. Microbiol.* 5, 939-951

Di Venanzio, G., Flores-Mireles, A. L., Calix, J. J., Haurat, M. F., Scott, N. E., et al., (2019). Urinary tract colonization is enhanced by a plasmid that regulates uropathogenic *Acinetobacter baumannii* chromosomal genes. *Nat. Commun*. 10, 2763. DOI: 10.1038/s41467-019-10706-y

Eveillard, M., Soltner, C., Kempf, M., Saint André, J. P., Lemarié, C., et al., (2010). The virulence variability of different *Acinetobacter baumannii* strains in experimental pneumonia. *J. Infect.* 60, 154-161. DOI: 10.1016/j.jinf.2009.09.004

Falagas, M. E. and Rafailidis, P. I. (2009). Nephrotoxicity of colistin: new insight into an old antibiotic. *Clin. Infect. Dis.* 48, 1729-1731. DOI: 10.1086/599226

Fragkou, P. C., Poulakou, G., Blizou, A., Blizou, M., Rapti, V., et al., (2019). The Role of minocycline in the treatment of nosocomial infections caused by multidrug, extensively drug and pandrug resistant *Acinetobacter baumannii*. *Syst Rev Clin. Evid. Microorg.* 7, 159. DOI: 10.3390/microorganisms7060159

Garnacho-Montero, J., Amaya-Villar, R., Ferrándiz-Millón, C., Díaz-Martín, A., López-Sánchez, J. M. and Gutiérrez-Pizarraya, A. (2015). Optimum treatment strategies for carbapenem-resistant *Acinetobacter baumannii* bacteremia. *Expert Rev. Anti. Infect. Ther*. 13, 769-777. DOI: 10.1586/14787210.2015.1032254

Inci, A., Toker, M. K., Bicer, I. G., Derbent, A. and Salihoglu, Z. (2018). Determination of colistin-related nephrotoxicity and risk factors in intensive care unit. *North Clin. Istanb*. 5, 120-124. DOI: 10.14744/nci.2017.42243

Lee, C. R., Lee, J. H., Park, M., Park, K. S., Bae, I. K., et al., (2017). Biology of *Acinetobacter baumannii*: Pathogenesis, antibiotic resistance mechanisms, and prospective treatment options. *Front. Cell. Infect. Microbiol*. 7, 55. DOI: 10.3389/fcimb.2017.00055

Long, W., Yuan, J., Liu, J., Liu, J., Wu, M., et al., (2018). Multidrug resistant brain abscess due to *Acinetobacter baumannii* ventriculitis cleared by intraventricular and intravenous tigecycline therapy: A Case report and review of literature. *Front. Neurol*. 9, 518. DOI: 10.3389/fneur.2018.00518

Morris, F. C., Dexter, C., Kostoulias, X., Uddin, M. I., and Peleg, A. Y. (2019). The Mechanisms of Disease Caused by *Acinetobacter baumannii*. *Front Microbiol*. 10, 1601. Published online 2019 Jul 17. doi: 10.3389/fmicb.2019.01601 PMCID: PMC6650576 ArticlePubReaderPDF–1.9MCitation

Peleg, A. Y., Adams, J. and Paterson, D. L. (2007). Tigecycline efflux as a mechanism for nonsusceptibility in *Acinetobacter baumannii*. *Antimicrob. Agents Chemother*. 51, 2065-2069. DOI: 10.1128/AAC.01198-06

Pfalzgraff, A., Brandenburg, K. and Weindl, G. (2018). Antimicrobial Peptides and Their Therapeutic Potential for Bacterial Skin Infections and Wounds. *Front Pharmacol.* 9, 281. doi: 10.3389/fphar.2018.00281. eCollection 2018. Review. PMID: 29643807 Free PMC Article

Serota, D. P., Sexton, M. E., Kraft, C. S. and Palacio, F. (2018). Severe community-acquired pneumonia due to *Acinetobacter baumannii* in North America: Case report and review of the literature. *Open Forum Infect*. *Dis*.

de Breij, A., Dijkshoorn, L., Lagendijk, E., van der Meer, J., Koster, A., Bloemberg, G., et al., (2010). Do biofilm formation and interactions with human cells explain the clinical success of *Acinetobacter baumannii*? *PLoS One* 5:e10732. DOI: 10.1371/journal.pone.0010732

de Breij, A., Eveillard, M., Dijkshoorn, L., van den Broek, P. J., Nibbering, P. H. and Joly-Guillou, M. L. (2012). Differences in *Acinetobacter baumannii* strains and host innate immune response determine morbidity and mortality in experimental pneumonia. *Plos One.* 7:e30673. DOI: 10.1371/journal.pone.0030673

Casallas, J. C. G., Robayo-Amortegui, H., Corredor-Rozo, Z., Carrasco-Márquez, A. M. and Escobar-Perez, J. (2019). Bacteremia by colistin-resistant *Acinetobacter baumannii* isolate: a case report. *J. Med. Case Rep*. 13, 141. DOI: 10.1186/s13256-019-2062-3

Ceylan, B., Arslan, F., Sipahi, O. R., Sunbul, M., Ormen, B., et al., (2017). Variables determining mortality in patients with *Acinetobacter baumannii* meningitis/ventriculitis

treated with intrathecal colistin. *Clin. Neurol. Neurosurg*. 153, 43-49. DOI: 10.1016/j.clineuro.2016.12.006

Chen, Q., Cao, H., Lu, H., Qiu, Z. H. and He, J. J. (2015). Bioprosthetic tricuspid valve endocarditis caused by *Acinetobacter baumannii* complex, a case report and brief review of the literature. *J. Cardiothorac. Surg*. 10, 149. DOI: 10.1186/s13019-015-0377-8

Chim, H., Tan, B. H. and Song, C. (2007). Five-year review of infections in a burn intensive care unit: High incidence of *Acinetobacter baumannii* in a tropical climate. *Burns* 33, 1008-1014. DOI: 10.1016/j.burns.2007.03.003

Daskalaki, E., Koukoulaki, M., Bakalis, A., Papastamopoulos, V., Belesiotou, E., et al., (2014). Blood stream infections in renal transplant recipients: a single-center study. *Transplant Proc*. 46, 3191-3193. DOI: 10.1016/j.transproceed.2014.10.033

Dexter, C., Murray, G. L., Paulsen, I. T. and Peleg, A. Y. (2015). Community-acquired *Acinetobacter baumannii*: clinical characteristics, epidemiology and pathogenesis. *Expert Rev. Anti. Infect. Ther*. 13, 567-753. DOI: 10.1586/14787210.2015.1025055

Dijkshoorn, L., Nemec, A. and Seifert, H. (2007). An increasing threat in hospitals: multidrug resistant Acinetobacter baumannii. *Nat. Rev. Microbiol.* 5, 939-951.

Di Venanzio, G., Flores-Mireles, A. L., Calix, J. J., Haurat, M. F., Scott, N. E., et al., (2019). Urinary tract colonization is enhanced by a plasmid that regulates uropathogenic *Acinetobacter baumannii* chromosomal genes. *Nat. Commun*. 10, 2763. DOI: 10.1038/s41467-019-10706-y

Eveillard, M., Soltner, C., Kempf, M., Saint André, J. P., Lemarié, C., et al., (2010). The virulence variability of different *Acinetobacter baumannii* strains in experimental pneumonia. *J. Infect.* 60, 154-161. DOI: 10.1016/j.jinf.2009.09.004

Falagas, M. E. and Rafailidis, P. I. (2009). Nephrotoxicity of colistin: new insight into an old antibiotic. *Clin. Infect. Dis.* 48, 1729-1731. DOI: 10.1086/599226

Fragkou, P. C., Poulakou, G., Blizou, A., Blizou, M., Rapti, V., et al., (2019). The Role of minocycline in the treatment of nosocomial infections caused by multidrug, extensively drug and pandrug resistant *Acinetobacter baumannii*. *Syst Rev Clin. Evid. Microorg.* 7, 159. DOI: 10.3390/microorganisms7060159

Garnacho-Montero, J., Amaya-Villar, R., Ferrándiz-Millón, C., Díaz-Martín, A., López-Sánchez, J. M. and Gutiérrez-Pizarraya, A. (2015). Optimum treatment strategies for carbapenem-resistant *Acinetobacter baumannii* bacteremia. *Expert Rev. Anti. Infect. Ther*. 13, 769-777. DOI: 10.1586/14787210.2015.1032254

Inci, A., Toker, M. K., Bicer, I. G., Derbent, A. and Salihoglu, Z. (2018). Determination of colistin-related nephrotoxicity and risk factors in intensive care unit. *North Clin. Istanb*. 5, 120-124. DOI: 10.14744/nci.2017.42243

Lee, C. R., Lee, J. H., Park, M., Park, K. S., Bae, I. K., et al., (2017). Biology of *Acinetobacter baumannii*: Pathogenesis, antibiotic resistance mechanisms, and prospective treatment options. *Front. Cell. Infect. Microbiol*. 7, 55. DOI: 10.3389/fcimb.2017.00055

Long, W., Yuan, J., Liu, J., Liu, J., Wu, M., et al., (2018). Multidrug resistant brain abscess due to *Acinetobacter baumannii* ventriculitis cleared by intraventricular and intravenous tigecycline therapy: A Case report and review of literature. *Front. Neurol*. 9, 518. DOI: 10.3389/fneur.2018.00518

Morris, F. C., Dexter, C., Kostoulias, X., Uddin, M. I., and Peleg, A. Y. (2019). The Mechanisms of Disease Caused by *Acinetobacter baumannii*. *Front Microbiol*. 10, 1601.

Published online 2019 Jul 17. doi: 10.3389/fmicb.2019.01601 PMCID: PMC6650576 ArticlePubReaderPDF–1.9MCitation

Peleg, A. Y., Adams, J. and Paterson, D. L. (2007). Tigecycline efflux as a mechanism for nonsusceptibility in *Acinetobacter baumannii*. *Antimicrob. Agents Chemother*. 51, 2065-2069. DOI: 10.1128/AAC.01198-06

Pfalzgraff, A., Brandenburg, K. and Weindl, G. (2018). Antimicrobial Peptides and Their Therapeutic Potential for Bacterial Skin Infections and Wounds. *Front Pharmacol.* 9, 281. doi: 10.3389/fphar.2018.00281. eCollection 2018. Review. PMID: 29643807 Free PMC Article

Serota, D. P., Sexton, M. E., Kraft, C. S. and Palacio, F. (2018). Severe community-acquired pneumonia due to *Acinetobacter baumannii* in North America: Case report and review of the literature. *Open Forum Infect*. *Dis*.
